# Chromosome folding and prophage activation reveal specific genomic architecture for intestinal bacteria

**DOI:** 10.1186/s40168-023-01541-x

**Published:** 2023-05-19

**Authors:** Quentin Lamy-Besnier, Amaury Bignaud, Julian R. Garneau, Marie Titecat, Devon E. Conti, Alexandra Von Strempel, Marc Monot, Bärbel Stecher, Romain Koszul, Laurent Debarbieux, Martial Marbouty

**Affiliations:** 1 Institut Pasteur, Université Paris Cité, CNRS UMR6047, Bacteriophage Bacterium Host, 25-28 Rue du Dr Roux, 75015 Paris, France; 2 Institut Pasteur, Université Paris Cité, Spatial Regulation of Genomes Group, CNRS UMR 3525, 25-28 Rue du Dr Roux, 75015 Paris, France; 3 Sorbonne Université, Collège Doctoral, Paris, France; 4 Institut Pasteur, Université Paris Cité, Plate-Forme Technologique Biomics, 75015 Paris, France; 5 Université de Lille, INSERM, CHU Lille, U1286-INFINITE-Institute for Translational Research in Inflammation, Lille, 59000 France; 6 Max Von Pettenkofer Institute of Hygiene and Medical Microbiology, Faculty of Medicine, LMU Munich, Munich, Germany; 7 German Center for Infection Research (DZIF), Partner Site LMU Munich, Munich, Germany

**Keywords:** Phages, Gut, HiC, Virome, OMM12, 3D signatures

## Abstract

**Background:**

Bacteria and their viruses, bacteriophages, are the most abundant entities of the gut microbiota, a complex community of microorganisms associated with human health and disease. In this ecosystem, the interactions between these two key components are still largely unknown. In particular, the impact of the gut environment on bacteria and their associated prophages is yet to be deciphered.

**Results:**

To gain insight into the activity of lysogenic bacteriophages within the context of their host genomes, we performed proximity ligation-based sequencing (Hi-C) in both in vitro and in vivo conditions on the 12 bacterial strains of the OMM^12^ synthetic bacterial community stably associated within mice gut (gnotobiotic mouse line OMM^12^). High-resolution contact maps of the chromosome 3D organization of the bacterial genomes revealed a wide diversity of architectures, differences between environments, and an overall stability over time in the gut of mice. The DNA contacts pointed at 3D signatures of prophages leading to 16 of them being predicted as functional. We also identified circularization signals and observed different 3D patterns between in vitro and in vivo conditions. Concurrent virome analysis showed that 11 of these prophages produced viral particles and that OMM^12^ mice do not carry other intestinal viruses.

**Conclusions:**

The precise identification by Hi-C of functional and active prophages within bacterial communities will unlock the study of interactions between bacteriophages and bacteria across conditions (healthy vs disease).

Video Abstract

**Supplementary Information:**

The online version contains supplementary material available at 10.1186/s40168-023-01541-x.

## Background

Microbial communities that inhabit the mammal intestinal tract are complex and predominantly composed of a variety of bacteria and their specific viruses, the bacteriophages (phages). Variations in the composition of both bacterial and viral intestinal communities have been associated with a wide range of diseases [1–4]. However, the mechanisms supporting these associations are still poorly understood. Indeed, the broad diversity of intestinal microbes residing in the gut of humans or laboratory animals hinders the precise identification of the role of each component.

Over the last decade, multiple studies have employed proximity ligation-based technologies [5] (e.g., Hi-C) to study the 3D organization of bacterial genomes [6–11]. These approaches, which quantify the relative collision frequencies between DNA segments, revealed the local and global folding of bacterial chromosomes. Using various growth conditions and mutants in combination with other omics approaches, these studies have allowed to better understand the role of nucleoid-associated proteins (NAPs) and transcription in the 3D genome architecture of model bacteria and its structuration within chromosome interaction domains (CIDs) [6–8, 12, 13]. However, major clades of the intestinal microbial communities remain unexplored. Moreover, studies have only focused on in vitro cultures leaving open questions on the 3D chromosomal architectures in the digestive tract.

In parallel, we and others have developed Hi-C derivatives approaches to improve metagenome analysis (e.g., meta3C or metaHi-C) [14–16] (reviewed in [17]). Taking advantage of DNA contacts as a marker of relative physical proximity, metaHi-C was developed and applied to successfully infer the bacterial hosts of episomes, such as plasmids and phages, in complex natural microbial populations [14, 18–21]. We also demonstrated the possibility of using Hi-C data to study DNA segment integration in bacterial genomes as well as prophages through the detection of 3D signatures [8, 14, 19].

Gnotobiotic animals, in which a defined population of microbes is introduced, provide a model to apply our approaches on semi-controlled communities. In 2016, the mouse line named Oligo-MM12 (OMM^12^) was proposed to the community as a platform from which the role of individual bacteria and environmental parameters (e.g., diet, infection) could be investigated with high reproducibility [22]. These 12 strains were initially isolated from conventional mice intestines and are representative of the five most abundant phyla in the mouse gut microbiota: *Bacteroidetes*,* Firmicutes*,* Actinobacteria*,* Proteobacteria*, and *Verrucomicrobia*. A comparison of four different breeding facilities demonstrated negligible variations in the composition and relative abundance of these gut species [23]. Another advantage of this model is the possibility to track the genomic information over time as the 12 strains have been sequenced and assembled [24, 25]. Indeed, a long-term study, under constant environmental and nutritional conditions over several years, demonstrates a slow and progressive evolution of microbial sub-strains through positive selection [26], making it a suitable system to perform an in-depth analysis of the 3D organization of bacterial chromosomes within the intestinal community.

In the present study, we combined Hi-C with virome sequencing to characterize the 3D organization and behavior of chromosomes of bacteria of the OMM^12^ consortium as well as their associated (pro)phages in vitro but also in vivo (i.e., in the gut environment). Hi-C contact maps of the 12 individual bacteria (in vitro) confirm the central role played by the ParABS system in bacterial chromosome folding across new clades but also revealed an unexpected diversity of genome architectures of bacteria for which chromosome folding was never assessed before. Comparison with genome-wide contact maps of the same bacteria obtained from in vivo gut conditions showed that the overall chromosome folding is conserved with, nonetheless, several notable differences. The analysis of the Hi-C data detected 16 functional prophages exhibiting CID-like signatures out of 44 putative prophages predicted bioinformatically. It then allowed us to refine the coordinates of their borders and identified circularization events as well as their induction status. The concurrent virome sequencing of the samples showed that 11 of these 16 prophages formed virions, which we deeply characterized*.* We also found differences in the induction of the prophages between the two conditions (in vitro vs in vivo*)*. Finally, the same analyses performed 1 year apart revealed that despite some genetic diversity functional prophages were induced in a similar way, showing that they constitute a resident phage population in OMM^12^ mice gut.

## Methods

### Bacterial culture

The OMM^12^ strains originate from the miBC collection [27]. All bacteria were grown in anaerobic akkermansia medium (18.5 g.L^−1^ brain heart infusion, 15 g.L^−1^ trypticase soy broth, 5 g.L^−1^ yeast extract, 2.5 g.L^−1^ K_2_HPO_4_, 0.5 g.L^−1^ cysteine hydrochloride, 0.5 g.L^−1^ glucose, 0.4 g.L^−1^ Na_2_CO_3_, 1 mg.L^−1^ hemin, 0.5 mg.L^−1^ menadione, and 3% fetal calf serum, completement-inactivated, in distilled water) in an anaerobic chamber (1.5–3% H_2_, 4% CO_2_, rest N_2_). A 60 mL of medium was inoculated to reach a OD_600nm_ of 0.01 for each bacterium and were incubated without agitation at 37 °C. *Muribaculum intestinale* (YL27), *Clostridium innocuum* (I46), *Blautia coccoides* (YL58), *Limosilactobacillus reuteri* (I49), *Enterococcus faecalis* (KB1), and *Bifidobacterium longum *subsp*. animalis* (YL2) were cultured for 5 h, while *Bacteroides caecimuris* (I48), *Akkermansia muciniphila* (YL44), *Enterocloster clostridioformis* (YL32), *Flavonifractor plautii* (YL31), *Turicimonas muris* (YL45), and *Acutalibacter muris* (KB18) were cultured for 10 h. Half of each culture was centrifuged (6000 g, 15 min, 4 °C). The supernatant was frozen at − 80 °C. Formaldehyde was added to the other half (3% final), and the mixture was incubated under gentle agitation at room temperature for 30 min, then at 4 °C for 30 min. A 5 mL of glycine 2.5 M was then added, followed by an incubation at room temperature for 20 min under gentle agitation. The solution was centrifuged (6000 g, 10 min, 4 °C), and the pellet was washed in 1X PBS. After a similar centrifugation, the supernatant was removed, and the pellet was frozen at − 80 °C.

### Hi-C library generation

Hi-C libraries were generated as previously described [20]. Mice fecal samples were collected and directly mixed in 10 mL of crosslinking solution (1X PBS supplemented with 3% formaldehyde) and incubated for 1 h at room temperature under strong agitation. Formaldehyde was quenched by adding 5 mL of 2.5 M glycine for 20 min at room temperature under gentle agitation. The samples were then recovered by centrifugation (6000 g, 10 min, 4 °C), washed with 10 mL 1X PBS, re-centrifuged, and stored at − 80 °C until processing. Each sample was resuspended in 1.2 mL TE 1X supplemented with antiprotease (mini tablets—Roche), transferred in a Precelys tubes (2 mL—VK05 supplemented with 100 µL of VK01 glass beads), and disrupted (6700 rpm—20 s ON/30 s OFF—6 cycles). Lysates were recovered and SDS 10% was added to a final concentration of 0.5%, and the lysate was incubated for 10 min at RT. For each library, 1 mL of lysate was transferred to a tube containing the digestion reaction solution (500 µL NEB1 10X buffer, 500 µL Triton 10%, 1000 U Sau3AI, H_2_O, final volume = 4 mL). Digestion was allowed to proceed for 3 h at 37 °C under gentle agitation. Tubes were then centrifuged for 20 min at 4 °C and 16,000 g, the supernatants were discarded, and pellets were resuspended in 400 µL H_2_O. Biotinylation was done by adding 50 µL NEB ligation buffer 10X (without ATP), 4.5 µL of 10 mM dATP/dTTP/dGTP, 37.5 µL biotin-dCTP 0.4 mM, and 8 µL Klenow (5U/µL). Reactions were incubated for 45 min at 37 °C and then transferred to a tube containing the ligation reaction (160 µL NEB ligation buffer 10X, 16 µL ATP 100 mM, 16 µL BSA 10 mg/mL, 500 U T4 DNA ligase, final volume = 1.1 mL). Ligations were processed for 3 h at RT. 20 µL EDTA 0.5 M, 80 µL SDS 10%, and 2 mg proteinase K were added to each reaction and incubated overnight at 65 °C to digest proteins. DNA was extracted using phenol–chloroform and precipitated with 2.5 × vol. ethanol 100%. Pellets were suspended in a final volume of 130 µL TE 1X supplemented with RNAse, incubated for 1 h at 37 °C, and stored at − 20 °C until use. DNA was extracted, purified, and processed into a sequencing library as described previously [28]. Proximity ligation libraries were sequenced using pair-end (PE) Illumina sequencing (2 × 35 bp, NextSeq500 apparatus) (Supplementary Table 1).

### Hi-C analysis

Forward and reverse reads were aligned separately with bowtie2 v2.3.5.1 [29]. From these alignments, Hi-C matrices and genomic distance law were generated using hicstuff v3.0.3 (https://github.com/koszullab/hicstuff). Matrices were balanced using the ICE algorithm [30]. For comparative analysis, matrices were binned at 10 kbp resolution and were downsampled to the same number of contacts. Reproducibility between replicates was assessed using the hicreppy v0.0.6 implementation (https://github.com/cmdoret/hicreppy) of the HiCrep algorithm [31]. Comparison between matrices was done using log2 ratio and serpentine v0.1.3 60 for flexible binning [32].

### Bacterial genome annotations and genomic features

Genomes were annotated using the last version of the NCBI Prokaryotic Genome Annotation Pipeline (PGAP) [33]. The coverage was computed using tinycov (https://github.com/cmdoret/tinycov), v0.3.0. The GC content and the GC skew were computed using dnaglider (https://github.com/cmdoret/dnaglider), v0.0.4. The *parS* sites have been detected using a degenerated *par*S consensus sequence TGTTTCACGTGAAACA [34] and allowing 2 mismatches. The ori and ter positions were approximated based on the GC skew inversion. The shift closer to the *par*S cluster was annotated as the ori and the shift as the opposite of the genome as the ter. Figures were generated using pyGenometracks v3.6 [35].

### Prophage annotations

Prophage annotation was performed using the OMM^12^ genomes [25] except for *B. caecimuris*, *B. longum*, and *F. plautii* for which the version re-assembled in this article was used. Both Vibrant [36], v1.2.1 and Virsorter2 [37], v2.2.3 with their respective databases, were used to annotate the bacterial genomes. The data from mitomycin C-induced prophages was obtained from Zünd et al. [38]. Refinement of the prophage annotation using the HiC contact map was performed using an insulation score method [13] to detect the borders of the prophage region. We refine the borders of the in silico annotated prophage boundaries using the two closest detected borders. The code is available at https://github.com/abignaud/oligomm_analysis.

### Virome preparation

Fecal samples (2 pellets minimum) were collected and directly frozen at − 20 °C. The pellets were then resuspended in 14 mL Tris 10 mM, pH 8, and centrifuged for 10 min at 5200 g, 4 °C. A known number of phages were then added as a spike as indicated in Supplementary Table 1. The supernatant was then filtered (0.45 µm and 0.22 µm) and ultracentrifuged for 3 h at 270,000 g. The resulting pellet was resuspended in 500 µL of TN buffer (10 mM Tris, 150 M, pH 7.5). To remove free DNA and RNA, 4 U of Turbo DNaseI (Ambion) and 10 µL of RNAse (A/T1 mix, ThermoFisher) were added for 30 min at 37 °C. The DNAse was then inactivated with 15 mM final EDTA and treated with 100 µg/mL final of proteinase K (Eurobio) and 0.5% final SDS for 30 min at 55 °C. The viral DNA was extracted by adding a volume of phenol–chloroform-isoamyl alcohol (25:24:1), vortexing for 30 s, and centrifuging for 5 min at 12,000 g. The aqueous phase was recovered and treated again with phenol–chloroform-isoamyl alcohol similarly. The recovered DNA was precipitated with sodium acetate (300 mM final) and two volumes of 100% EtOH. 1 µL of glycogen was also used as a DNA carrier. The sample was mixed by inversion and incubated for 2 h at − 80 °C before centrifugation for 20 min at 15,000 g, 4 °C. The pellet was dried and resuspended in 20 µL Tris 10 mM, pH 8. The DNA concentration was measured with Qubit (Invitrogen). The dsDNA libraries were prepared using TruSeq Nano DNA Sample Preparation Kit (Illumina) and ssDNA/dsDNA libraries with the Accel-NGS™ 1S Plus DNA Library Kit (Swift Biosciences). They were sequenced on a NextSeq550 or Novaseq (Illumina) for the 2 × 35 nt libraries and on a MiSeq (Illumina) for the 2 × 150 libraries (Supplementary Table 1).

The supernatant of individual OMM^12^ strain cultures was filtered (0.45 µm and 0.22 µm) and ultracentrifuged for 3 h at 270,000 g. The following steps were like the treatment of fecal samples described above. The DNA obtained for each strain was mixed in a single sequencing run, on a MiSeq (Illumina) with a 2 × 150 library.

### Annotation of induced prophages

The quality of the reads was assessed with FastQC (https://www.bioinformatics.babraham.ac.uk/projects/fastqc/). The reads were cleaned with cutadapt v2.10 [39] using a quality threshold of 20 and a minimum size of 30 for 35 bp reads or 140 for 150 bp reads. Using all the virome libraries, the precise coordinates of the prophages were determined by visualizing individual reads on IGV [40] v2.11.9 and looking for paired-end reads mapping both at different locations of the bacterial genome in areas bioinformatically predicted as prophage regions. The observed circularization signal indicated the production of viral particles and thus the coordinates of the phage genome.

Using those coordinates, the precise sequence of each prophage was extracted and annotated as follows. A first annotation was realized using PATRIC [41], with the “phage” recipe. Then, each predicted protein was compared to the PHROG [42] database v3 using the HHsuite v3.3.0 (hhsearch_omp function). For hits with a probability above 88%, the corresponding PHROG annotation was manually added to the corresponding protein. The annotated genomes were then visualized using Clinker [43] v1.1.0.

### RPKM counts of induced prophage regions in OMM^12^ virome samples

The virome reads were mapped using bowtie2 [29] v2.3.5.1 with default parameters against the most recent version of each of the 12 reference genomes of the OMM^12^, except for *B. caecimuris*, *F. plautii*, and *B. animalis* for which the version attached to this article was used. The number of reads mapping on each prophage was divided by the number of reads (per million) and by the length of the prophage region (per kb), resulting in RPKM counts for each sample and prophage region.

In order to estimate the number of VLP present in the virome samples, the virome reads were similarly mapped on the genome of the spiked phages (CLB_P1: KC109329, CLB_P2: OL770107, CLB_P3: OL770108, M13: NC_025824). RPKM counts were similarly calculated and compared to the concentration of phage spike in the corresponding sample (Supplementary Table 1) to obtain the estimation of the number of VLP present in OMM^12^ fecal samples. This value was then divided by the initial weight of the fecal sample used for the virome protocol to obtain VLP/g values. A value of 2.47 × 10^8^ PFU/g was obtained for Virome 7 and 1.09 × 10^8^ PFU/g for Virome 8.

### Prediction of phage termini and packaging mechanism

PhageTermVirome (PTV) [44] v.4.0.0 was run using virome sequencing reads that mapped to each inducible prophage region. PTV was run in paired-end mode, with seed parameter -s 15 and peak merging parameter -d 8. Packaging mechanisms were predicted both by statistical analysis and visual confirmation of coverage patterns near termini.

### Shared-protein network analysis

OMM^12^-induced prophages were clustered using vConTACT2 [45] v0.9.19 with the following parameters: –rel-mode 'Diamond' –db 'ProkaryoticViralRefSeq201-Merged' –pcs-mode MCL –vcs-mode ClusterONE. Publicly available phage genomes from ViralRef Seq V.201 [46] and Cenote Human Virome Database (CHVD) [47] were used as reference collections for the analysis. The resulting network was visualized and annotated using Cytoscape v3.9.1 [48]. The edge-weighted spring-embedded model was applied to position genomes sharing most protein clusters.

### Search for virulent phages in OMM^12^ fecal virome

Cleaned virome reads were mapped on both the genomes of the OMM^12^ strains and the Mus musculus genome (NC_000067.7). The non-mapping reads were then used to perform assemblies with MEGAHIT v1.2.9 [49] and SPAdes v3.15.2 [50], both with default parameters. The resulting contigs < 5 kb were discarded, and the other contigs were analyzed using PATRIC [41] and BLAST [51]. The same non-mapping reads were also analyzed by Kaiju v1.7.3 [52] in order to obtain taxonomic assignment. The results were visualized using Krona [53].

### Detection of ssDNA phage in virome samples

Cleaned reads from virome 5 and virome 6 (Supplementary Table 1) were merged into one single dataset. Reads were assembled using SKESA [54] v2.4.0 and Metaviral SPAdes [55] v3.15.2 using k-mer size 21 and resulting contigs (≤ 15 kb ≥ 1 kb) were analyzed with VIBRANT [36] v1.2.1 and VirSorter2 [37] v2.2.3 with a default option to predict ssDNA sequences. Except for the M13 spiked control, no ssDNA was identified in the sequenced samples. All contigs from the SKESA and Metaviral SPAdes assemblies were joined in a single multifasta file and proteins were predicted and annotated using PROKKA [56]. The whole set of predicted proteins was then screened with BlastP for Microviridae characteristic MCP and Rep proteins contained in the PHROG database [42] (v3, PHROG 514, and 713, respectively). No significant hits were obtained on the database using a *e* value threshold of 1 × 10^–3^.

## Results

### In vitro characterization of chromosome folding in individual OMM^12^ bacteria revealed common principles of bacteria genome architectures

**Fig. 1:**
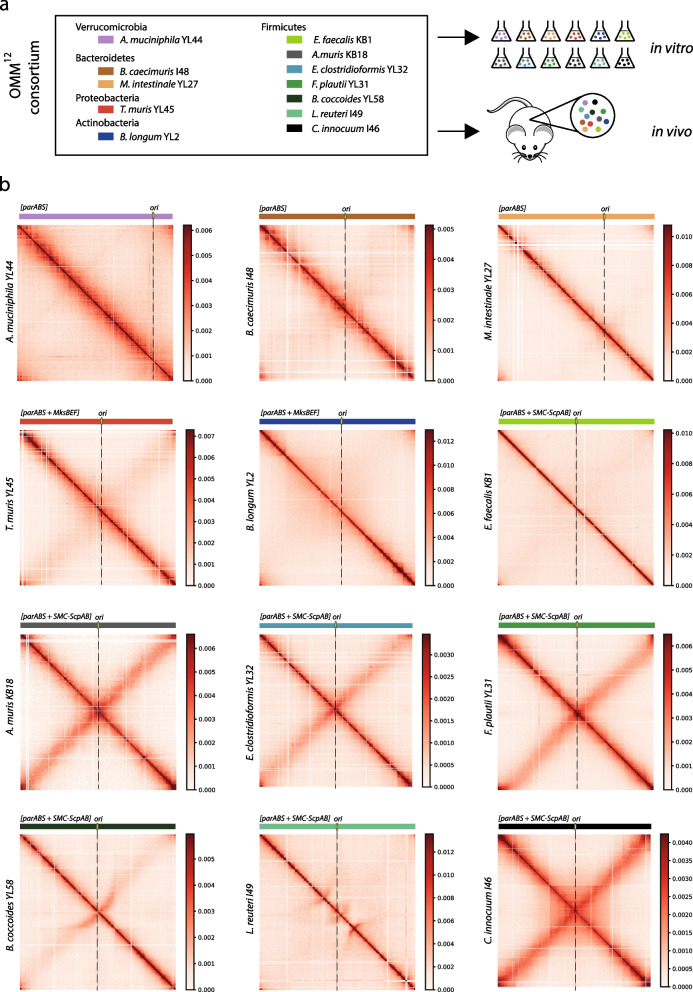
In vitro contact maps of the OMM^12^ consortium. **a** Experimental scheme of the source of Hi-C and virome libraries from the OMM^12^ bacteria. Hi-C and virome were performed both on independent in vitro cultures of each bacterium and from fecal samples. **b** Contact maps obtained from Hi-C performed on in vitro cultures for each bacterium of the OMM^12^ consortium (5 kb resolution). The localization of the origin of replication (*ori*) is indicated (black dashed line). Scale bars are indicated aside from each matrix

We applied a recently published high-resolution version of the Hi-C protocol adapted to bacteria to generate genome-wide contact maps for each of the 12 bacterial strains of the OMM^12^ consortium (Fig. 1, Supplementary Fig. 1, Supplementary Table 1 and Supplementary Table 2) (Methods, [57]). In model bacteria species, these contact maps typically display shared characteristics. First, a strong and broad diagonal reflects frequent local contacts between neighboring loci. This property can be exploited to scaffold incomplete genomes (reviewed in [58]) and was recently applied to close nine genomes of the OMM^12^ consortium [25]. Three genomes of the consortium (*B. caecimuris*,* B. animalis*, and *F. plautii*) remained nevertheless incomplete and were therefore scaffolded using the last published sequences (Supplementary Fig. 2). Next, bacterial contact maps typically exhibit self-interacting domains, the so-called Chromatin Interaction Domains or CIDs, visualized as squares along the main diagonal [6–10], whose boundaries correlate with high transcriptional activity and protein occupancy [6, 7]. As expected, CIDs were also found in the 12 contact maps, ranging in size from tens to hundreds of kilobases as previously observed for other bacteria (Supplementary Fig. 1) and with many boundaries colocalizing with transcriptionally active rRNA or tRNA loci. In addition, most bacteria studied so far (with the notable exception of *E. coli* [7] and to a lesser extent *V. cholerae* [10]) display a secondary diagonal perpendicular to the main one and extending from the origin of replication down to the terminus [8, 9, 59]. This pattern, which reflects enriched contacts between the two replichores along their entire length, was clearly observed for seven bacteria of the OMM^12^ (*B. caecimuris*,* T. muris*,* A. muris*,* E. clostridioformis*,* F. plautii*,* B. coccoides*,* C. innocuum*), barely visible in two (*A. muciniphila*,* E. faecalis*) and not detectable in the three others (*M. intestinale*,* B. longum*,* L. reuteri*) (Fig. 1 and Supplementary Fig. 1). Interestingly, the different matrices generated also revealed the diversity of bacteria chromosomes architectures with various structures such as the bow shape signals observed in *B. coccoides* and *L. reuteri* contact map. The 12 matrices generated confirm common principles but also reveal a large diversity of bacteria chromosome folding.

### ParS sites as major drivers of ori domains folding

**Fig. 2:**
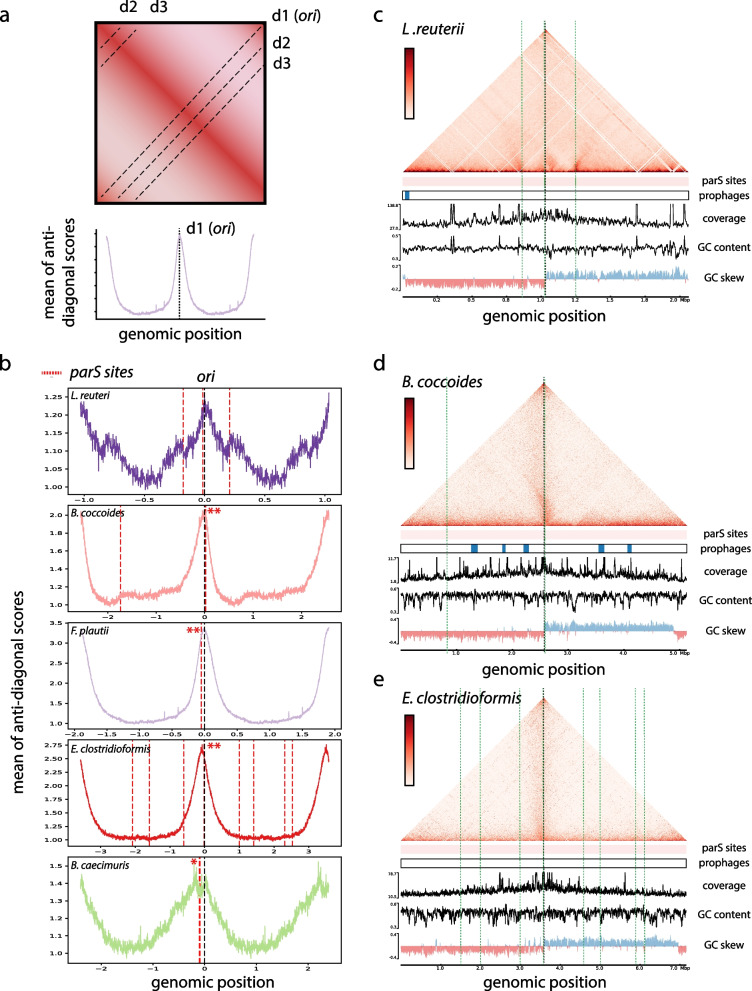
*parS* sites and their implications in the origin domain folding. **a** Schematic representation of a Hi-C contact map for a bacterial genome. The signal for each secondary diagonal (d1,d2,d3…) was computed in order to generate the graph presented below that showed the strength of the different secondary diagonals along the genome. Each graph was centered on the origin of replication. **b** Signal of the secondary diagonals for several OMM^12^ bacteria, centered on *ori* (*L. reuteri*,* B. coccoides*,* F. plautii*,* E. clostridioformis*,* B. caecimuris*). Localization of *parS* sites is indicated as red dashed lines. Red stars indicate the presence of several *parS* sites in the same 5 kb window. **c–e** The contact map of *L. reuteri* (5 kb resolution), *B. coccoides* (5 kb resolution), and *E. clostridioformis* (5 kb resolution). *ParS* sites (green dashed lines), putative prophages, coverage, GC content, GC skew, and genomic coordinates are indicated below each matrix

*ParS* sites are widely conserved [60] in bacteria and previous studies have demonstrated the central role of the ParABS system in regulating the overall 3D organization of bacterial genomes and in the segregation of their new replicated chromosomes [8, 11, 59, 61]. The number of *parS* sites varies considerably between bacteria ranging from one to 20 [34]. Former works have shown that the recruitment of the bacterial structural maintenance of chromosome (SMC) condensin complex SMC-ScpAB at *parS* sites generates hairpins and bridge replichores, through a loop-extrusion like mechanism [8, 62, 63]. We found homologs of the ParABS system in the 12 genomes confirming its large conservation in bacteria (Methods). Using *parS* consensus sequence, we detected between one and 10 *parS* sites in the 12 genomes with different distributions along the chromosome. In all cases, most of the detected *parS* sites are clustered and positioned at the crossing of the secondary diagonal (Fig. 2 and Supplementary Fig. 3). Using these *parS* clusters and the GC skew, we determined the position of the replication origin on the chromosomes (Methods). The read’s coverage variation is supported in all cases of these positions. Interestingly, the replication origin does not systematically correlate with the presence of the gene *dnaA*, which is typically used to define it. This suggests caution when using the *dnaA* gene in metagenomic studies to characterize contigs encompassing the origin of replication [64]. *ParS* clusters also associate with the presence of a large origin domain (*B. caecimuris*,* T. muris*,* A. muris*,* E. clostridioformis*,* F. plautii*,* C. innocuum*) or hairpin structures (*B. coccoides*,* L. reuteri*) (Supplementary Fig. 1) reminiscent of those observed for *B. subtilis* [8]. We further explored the possible link between *parS* sites, their numbers, their positions, and the presence of a secondary diagonal but could not detect any correlation with the strength of the signal in the opposite diagonal (Fig. 2b). The case of *L. reuteri* is particular in that we detect three sites that are quite distant to each other but still surround the origin of replication (*parS*1 =  − 180 kb, *parS*2 =  − 17 kb, *parS*3 =  + 206 kb) and, each time, are associated with a discrete 3D contact pattern (Fig. 2c). The proximal *parS*2 site is at the center of a small topological domain while the two other sites are linked with the typical hairpin signature observed in *B. subtilis* [8]. This specific structure of origin organization could be the result of the large distance separating the three *parS* sites in *L. reuteri*. Our data highlights the impact of *parS* site distribution in the overall folding of bacterial chromosomes.

### Bacteroides rely on currently unidentified SMC complexes for chromosomal arm alignment

Hi-C studies have shed light on the role of the SMC condensin family proteins in maintaining chromosome architecture [6, 8, 11, 59, 61]. Three types of complexes structurally related to condensins have been identified in bacteria: Smc-ScpAB, MukBEF, and MksBEF (review in [65]). Smc-ScpAB is present in most bacteria and often works together with the ParABS system in order to fulfill proper chromosome segregation [60]. In contrast, MukBEF is restricted to *Enterobacteria* whereas MksBEF is scattered over the phylogenic tree and both systems do not appear to promote chromosomal arms juxtaposition [7, 59, 61]. Among the 12 genomes, seven belonging to the *Firmicutes* phylum contain a clear homolog of the Smc-ScpAB proteins and two (*B. longum* and *T. muris*) have a distant homolog of the MksBEF system. For three bacteria (*A. muciniphila*,* B. caecimuris*, and *M. intestinale*), including the two belonging to the phylum *Bacteroidetes*, no homologs of known bacterial condensins could be found (Methods). This observation suggests that this family of proteins is not ubiquitous in bacteria [66, 67] and that other possible distant homologs have yet to be identified. Two of the species encoding Smc-ScpAB homologs do not display a secondary diagonal in their genomic contact maps (*L. reuteri* and *E. faecalis*), indicating that the presence of the Smc-ScpAB system does not systematically lead to a tight bridging of chromosomes arms (Figs. 1 and 2). On the other hand, we detect in *B. caecimuris* and especially in *T. muris* a secondary diagonal signal despite no homologs of condensins are found in these genomes (Methods). We also detect weak homologs of MksBEF in *T. muris*, a complex that is not known to promote arm alignment [59].

Our data indicate that possible distant homologs of SMC proteins or even new processes involved in chromosomal arm alignment remain to be uncovered. This Hi-C experiment on various bacteria species, including several Verrucomicrobia and Bacteroides, opens new avenues regarding chromosome folding regulation in prokaryotes.

### Bacteria chromosomal architectures present variations but remain stable in the mice gut environment

**Fig. 3:**
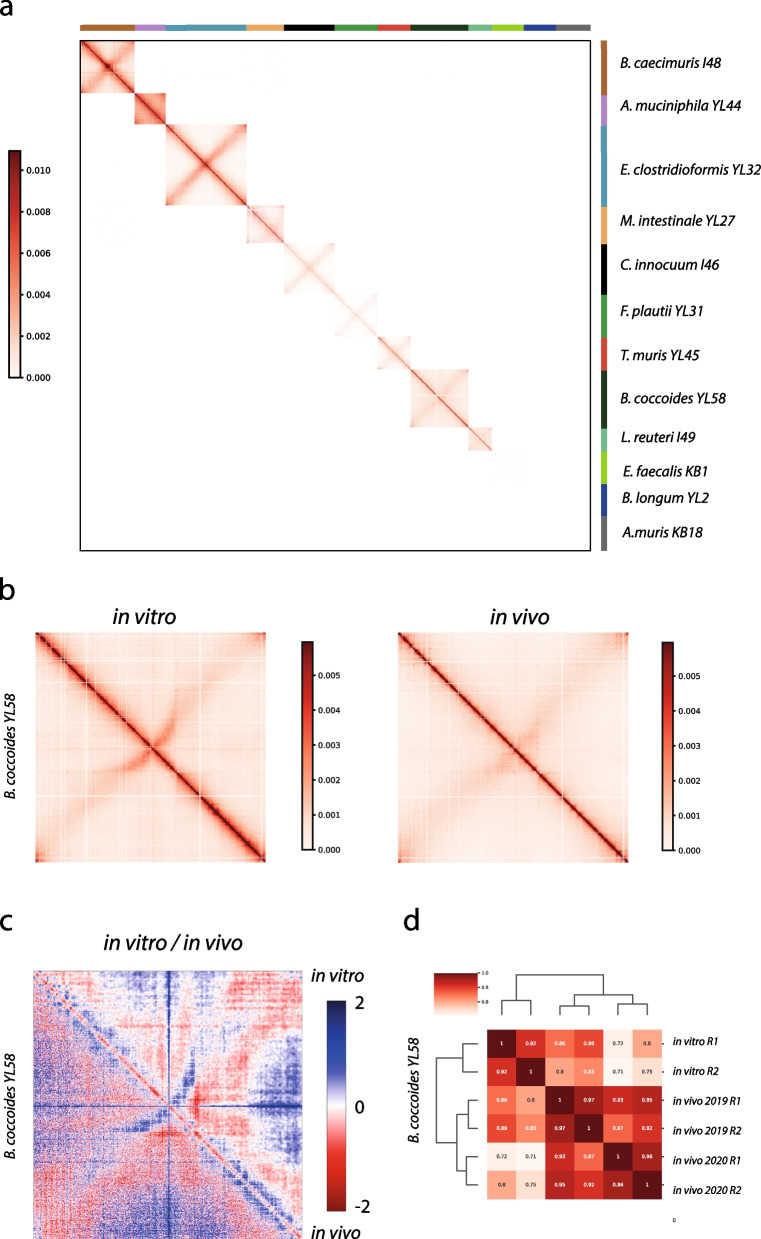
Stability of the chromosome architectures in the gut environment. **a** The contact map of the entire OMM^12^ consortium (5 kb resolution) obtained from fecal samples. The scale bar is indicated on the left and the different organisms on the right. **b** Comparison of the contact maps for *B. coccoides* between in vitro (left) and in vivo (right) conditions. **c** Ratio (Log2) of contact maps of the two maps of *B. coccoides* shown in panel **b**. An increase or decrease in contacts is represented in blue (in vitro) or red (in vivo*)*, respectively. **d** Hierarchical clustering of the different Hi-C replicates for *B. coccoides* using the software HiCrep

We next asked whether the chromosome organization of each bacterium grown individually is affected when they grow altogether in the gut of mice. We applied our latest metagenomic Hi-C protocol to mouse feces (*n* = 2; September 2019; Methods; [20]) and generated the resulting contact matrices for the entire consortium and for each bacterium (Fig. 3 and Supplementary Fig. 4). The resulting contact map shows no background signal between the different genomes, demonstrating the efficiency of the protocol (Fig. 3a). It also confirms that the genomes are well assembled, without contamination from one bacterium to another. Of the 12 bacteria present in the consortium, only six are sufficiently abundant to obtain individual contact matrices with exploitable signal (*B. caecimuris*, *E. clostridioformis*, *A. muciniphila*, *M. intestinale*, *C. innocum*, *B. coccoides*). Analysis of the different matrices obtained shows that large structures like the secondary diagonal signal were preserved in the intestinal environment while local structures exhibit important differences (Fig. 3b, c and Supplementary Fig. 4). For three species (*A. muciniphila*,* B. caecimuris*, and *E. clostridioformis*), we detected an increase of short-range contact in the in vivo conditions associated with a decrease of long-range interactions. For *C. innocuum* and *B. coccoides*, we observed an increase of the interactions at very short-range distance in the in vivo conditions. Finally, in *M. intestinale*, the contact map from the fecal sample shows notable differences compared to lab conditions with a clear increase of long-range interactions associated with low GC content loci. We also observed a portion of the genome exhibiting multiple long-range interactions, reminiscent of the loops observed in *B. subtilis* [8] and recently shown to correspond to Rok-dependant contacts, low GC content and recently acquired genetic material [68]. We could not detect specific annotations associated with this pattern, and further experiments and analysis will be needed to understand those differences. Our results were confirmed by performing Hi-C on feces sampled nine months later (*n* = 2; May 2020) from mice bred in the same facility. Comparison using HiCRep ([31]; Method) shows that single bacterium contact matrices from the two-time points are highly similar, demonstrating the stability of the observed 3D structures and the overall bacterial community (Fig. 3d and Supplementary Fig. 5). Although *M. instestinale* is less abundant in the second sample, the peculiar structures of its genome are still detected.

Differences in the 3D organization of genomes between in vitro and in vivo conditions highlight the impact of the intestinal tract on bacteria metabolism. Coupled with the stability of structures detected in vivo, it demonstrates that OMM^12^ mice can be further exploited to investigate chromosome architecture and dynamics in the gut environment.

### The 3D signatures of predicted prophage regions of OMM^12^ bacteria distinguish functional from cryptic prophages

**Fig. 4:**
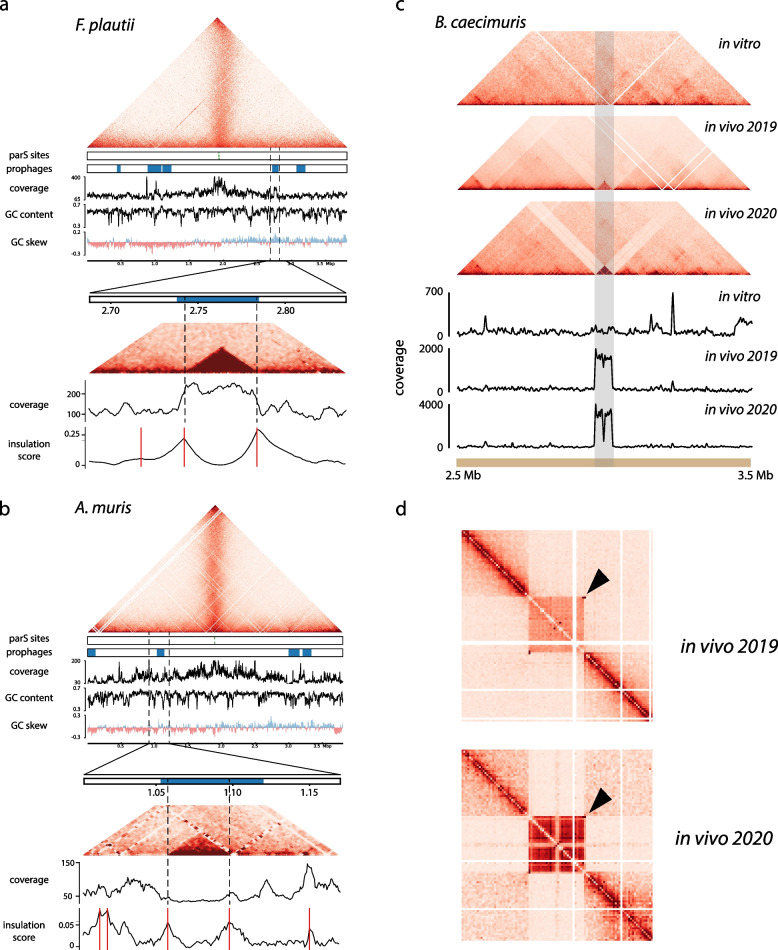
Contact maps and prophage regions in OMM^12^ bacteria. **a**,** b** Contact map of *F. plautii* and *A. muris*. The contact map is shown (5 kb resolution) on top, while additional information (localization of *parS* sites, putative prophage regions, coverage, GC content, GC skew, and genomic coordinates) is shown on the bottom. A zoom of the contact maps is visible on one putative prophage region per bacteria (2 kb resolution), with the associated coverage. Prophages are visible as red squares. Hi-C signal prediction is indicated in dashed lines. **c** Contact matrices (5 kb resolution) and associated coverage of *B. caecimuris* for in vitro and in vivo Hi-C matrices. The region corresponding to the induced prophages is highlighted in grey. **d** Zoomed contact matrix, centered around the induced prophage of *B. caecimuris* (2 kb resolution). The circularization signal is highlighted by a black arrow. The depleted region of the phage is visible in the middle of the square formed by the prophage

Analysis of the 12 bacterial genomes using VirSorter2 [37] and VIBRANT [36] revealed that no prophages was identified in *M. intestinale* and *B. animalis*, whereas a total of 44 prophages were predicted in the other 10 genomes, with 13 prophages in *E. clostridioformis* alone. Of these 44 candidate prophages, 14 exhibit a CID-like pattern (visible as a small square signal), allowing their coordinates to be refined using insulation score, a method similar to directional index [6] used to define CIDs borders [13] (Table 1, Fig. 4a, b and Supplementary Fig. 6, Methods). Interestingly, four prophages show coverage above the median coverage of the corresponding bacterial genome, which may indicate that they are induced (YL44-pp-0.038, YL44-pp-0.712, YL32-pp-3.355, and YL31-pp-2.738) (Table 1). We noticed that the *A. muciniphila* prophage (YL44-pp-0.038) has a typical profile of duplicated sequences that correspond to strong interactions with several loci, visible as stripes in the contact matrix, which could explain the difference in coverage. Such a pattern could be the result of phage propagation at different loci in the bacterial population. For the remaining 10 prophages, the absence of a clear differential coverage associated with the presence of a CID-like pattern could be the result of basal and non-abundant induction of the corresponding prophages. Interestingly, all six mitomycin C-induced prophages characterized previously by Zünd et al. [38] exhibit a clear signal showing that Hi-C enables reliably the detection of functional prophages without the need of specific inducing molecules. These results highlight the benefit of using Hi-C to characterize prophages induction in vitro, with the power to discriminate between functional and cryptic prophages and to refine precisely their boundaries. Other candidate prophages were not associated with CID-like pattern or loop signal potentially reflecting their non functionality.

**Table 1:** Candidate prophages of the OMM^12^ consortium

Bacteria		Localization (Mbp)	Vibrant	Virsorter2	3C in vitro	3C in vivo	3C in vitro vs in vivo	Virome
***A. muris***	***KB18***	0.026–0.095		Yes (Cat6)				
		**1.053–1.120**		Yes (Cat5)	Yes (TAD)	NA	NA	Yes
		2.999–3.151	Yes	Yes (Cat6)	Yes (TAD)	NA	NA	
		3.228–3.310		Yes (Cat5)				
***A. muciniphila***	***YL44***	0.038–0.072		Yes (Cat6)	Yes (TAD)	Yes (TAD)	Same	
		**0.712**–**0.743**	Yes	Yes (Cat5)	Yes (TAD)	Yes (TAD)	Same	Yes
		1.336–1.370	Yes	Yes (Cat6)				Yes
		**2.291**–**2.337**	Yes	Yes (Cat5)	Yes (depletion)	Yes (depletion)	Same	Yes
***B. caecimuris***	***I48***	0.271–0.355		Yes (Cat6)				
		0.922–1.008		Yes (Cat6)		Yes (TAD + loop)		
		1.409–1.421		Yes (Cat6)				
		2.969–3.118	Yes	Yes (Cat4)	Yes (loop + depletion)	Yes (TAD + loop)	Increased coverage and signal	Yes
***B. coccoides***	***YL58***	1.287–1.351		Yes (Cat5)				
		1.845–1.851	Yes					
		2.228–2.268		Yes (Cat6)				
		**3.571**–**3.620**	Yes	Yes (Cat6)	Yes(TAD + loop)	Yes (TAD + loop)	Same	Yes
		4.091–4.112	Yes					
***E. clostridioformis***	***YL32***	1.254–1.385	Yes					
		1.275–1.461		Yes (Cat5)				
		1.398–1.461	Yes					
		1.613–1.669	Yes	Yes (Cat5)				
		2.059–2.106	Yes	Yes (Cat4)	Yes (TAD + depletion signal)	Yes (TAD + loop)	Increased loop signal	Yes
		2.326–2.428		Yes (Cat6)				
		2.914–3.014	Yes	Yes (Cat5)				
		3.355–3.418	Yes	Yes (Cat5)	Yes (TAD)	Yes (TAD)	Decreased signal	Yes
		4.054–4.122		Yes (Cat6)				
		4.983–5.030	Yes					
		5.653–5.746		Yes (Cat6)				
		6.292–6.376		Yes (Cat6)				
		6.752–6.852		Yes (Cat6)				
***C. innocuum***	***I46***	0.454–0.499		Yes (Cat6)	Yes (TAD + loop)	NA	NA	
		3.744–3.787	Yes	Yes (Cat5)	Yes (TAD)	NA	NA	
		4.275–4.452	Yes	Yes (Cat5)	Yes (multiple TADs)	Yes (TAD)	2 different phages	Yes
***E. faecalis***	***KB18***	1.672–1.774		Yes (Cat6)				
		1.972–2.046		Yes (Cat6)				
		2.899–2.914	Yes					
***F. plautii***	***YL31***	0.460–0.466	Yes					
		**0.909**–**1.064**	Yes	Yes (Cat5)	Yes (TAD + loop)	NA	NA	Yes
		1.126–1.212	Yes	Yes (Cat5)				Yes
		**2.738**–**2.785**	Yes	Yes (Cat5)	Yes (TAD)	NA	NA	Yes
		3.093–3.179		Yes (Cat6)				
***L. reuteri***	***I49***	0.019–0.045	Yes					
***T. muris***	***YL45***	2.099–2.135		Yes (Cat6)				
		2.100–2.144	Yes	Yes (Cat5)				

The localization and size of the putative prophages as predicted by Vibrant and Virsorter are indicated. When Vibrant and Virsorter disagreed, the combined longest location was selected. In the localization column, boldface indicates induced prophages described by Zund et al. Detected 3D patterns as well as their variations between in vitro and in vivo conditions are indicated (dark gray = strong signal, light gray = weak signal). *NA* Not applicable (coverage is too low). Induced phages detected through virome sequencing are indicated in the last column

We next analyzed the in vivo Hi-C data. Among the 14 prophages listed above, six belong to bacteria without sufficient coverage and four exhibited a similar pattern between the two conditions. In contrast, four present significant increases in coverage and/or changes in pattern compared to in vitro contact maps (I48-pp-2.969, YL32-pp-2.059, YL32-pp-3.355, and I46-pp-4.275) (Table 1 and Supplementary Fig. 6). The prophage I48-pp-2.969 of *B. caecimuris* presents a strong increase in its coverage associated with a clear CID-like pattern, an isolation from the rest of the genome as well as a circularization signal (Fig. 4c) [8, 20], indicating a different physiological state associated with a putative higher induction of this prophage. Interestingly, we observe a sharp decrease of coverage in the middle of the prophage sequence, which could be due to mapping issues or suggests that the region is deleted when the phage is induced. In *E. clostridioformis*, the prophage YL32-pp-2.059 shows an increase in coverage associated with the emergence of a circularization signal, while prophage YL32-pp-3.355 exhibits the opposite pattern. These observations suggest that the gut environment switches the activation of these two prophages. Finally, prophage I46-pp-4.275 of *C. innocuum* displays a puzzling profile. Indeed, while only the left part of the prophage was observable in vitro as a topological domain, its right part appears as a domain under in vivo conditions. This suggests the presence of two functional prophages, as identified by the characterization of two distinct circularization signals when aligning virome reads to this region, with different behaviors in the two conditions. We also detected exclusively under in vivo conditions an additional functional prophage of *B. caecimuris* with a loop motif associated with the formation of a self-interacting domain (Table 1 and Supplementary Fig. 6). Therefore, the Hi-C data showed that in seven bacteria, a total of 16 regions annotated as prophages exhibit 3D signatures with differential intensity and patterns (CID-like, loop) between in vitro and in vivo conditions reflecting various activities of these genetic elements.

### Comprehensive characterization of the virome of the OMM^12^ mice

**Fig. 5:**
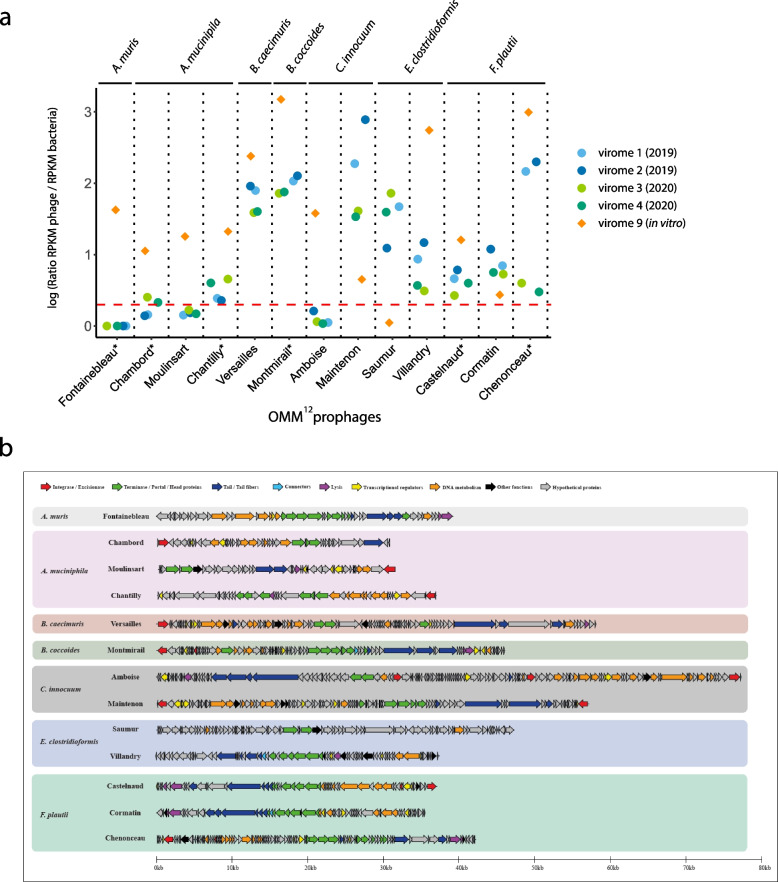
Active prophages of the OMM^12^ mice. **a** RPKM counts for the induced OMM^12^ prophages, for the different virome samples (Methods). Virome sequencing was performed for two fecal pellets of two mice in 2019 and 2020. The reads obtained were mapped on the 12 OMM.^12^ strains, and the resulting normalized sequencing depth is represented. Stars aside from prophages name under the graph indicate phages described by Zünd et al. The dashed red line indicates the induction threshold. **b** Genetic map of the 13 induced prophages. Prophages were grouped by host. Genes were colored based on predicted functions (Methods)

In order to evaluate to which extent the 3D patterns observed correlate with the production of phage particles, we performed virus-like particles (VLPs) sequencing (virome, see Methods) on the supernatants of in vitro and in vivo samples originating from the same cultures, the same cages and at the same time as the samples used for Hi-C (Supplementary Table 1). The reads obtained were mapped on the OMM^12^ genomes, and the resulting read per kilobase per million (RPKM) counts showed that multiple regions in the genomes of several strains contained significantly enriched counts, indicating that those regions are found in VLPs, and thus, presumably, correspond to induced prophages particles (Fig. 5a). Our data showed that a total of 13 prophages from seven strains were induced either in vitro (*n* = 12, including the six prophages previously identified by Zünd et al. [38]) and/or in vivo (*n* = 10). Out of the 16 prophages regions defined by Hi-C (15 in vitro and/or in vivo + one in vivo only), 11 were found to be among the 13 induced prophages. For the remaining five functional Hi-C-defined prophages, we were unable to detect any significant signal in the virome data suggesting that those prophages are not active enough, or not functional, or produce abortive cycles. Two induced prophages were also not found to be associated with a specific 3D pattern, possibly due to a lack of structuration of the corresponding genomic region or eventually to differences between fecal samples recovered for Hi-C and virome analyses. We then compared the prophages coordinates defined by Hi-C data with virome sequences and found that the boundaries of the prophages often (9/11) agree between the datasets (Table 2). These results clearly demonstrate that active and/or functional prophages present specific chromatin folding presumably through the activity of specific DNA binding proteins and/or transcriptional activity.

**Table 2:** Summary of the 13 induced prophages in the OMM^12^ consortium

Phage name	Fontainebleau*	Chambord*	Moulinsart	Chantilly*	Versailles	Montmirail*	Amboise	Maintenon	Saumur	Villandry	Castelnaud*	Cormatin	Chenonceau*
Bacteria	***A. muris***	***A. muciniphila***	***A. muciniphila***	***A. muciniphila***	***B. caecimuris***	***B. coccoides***	***C. innocuum***	***C. innocuum***	***E. clostridioformis***	***E. clostridioformis***	***F. plautii***	***F. plautii***	***F. plautii***
Virsorter2 coordinates	1,044,480–1,245,308	720,271–756,462	1,333,561–1,376,776	2,297,920–2,384,706	2,968,702–3,117,599	3,566,266–3,748,724	4,270,576–4,451,990	4,270,576–4,451,990	2,059,163–2,216,868	3,366,664–3,520,133	908,138–1,064,094	1,126,232–1,212,653	2,741,918–2,850,187
Vibrant coordinates	xxx	712,320–733,658	1,336,379–1,370,467	2,290,686–2,334,293	2,968,702–3,025,656	3,570,686–3,619,939	4,275,457–4,448,458	4,275,457–4,448,458	2,059,163–2,105,549	3,367,379–3,411,110	908,886–959,299	1,129,966–1,170,339	2,738,687–2,785,802
3C coordinates (kb)	1056–1098	711–724	xxx	2307–2324	2968–3027	3573–3616	4321–4399	4399–3	2065–2106	3375–3413	909–946	xxx	2742–2784
Virome coordinates	1,058,713–1,097,953	712,134–742,810	1,335,300–1,366,646	2,300,756–2,337,649	2,968,431–3,026,527	3,570,533–3,616,598	4,320,432–4,397,754	4,397,737–2635	2,059,120–2,106,361	3,375,269–3,412,801	908,701–945,825	1,128,657–1,164,096	2,742,851–2,784,974
3C Signal in vitro	Yes (strong)	Yes (strong)	No	Yes (weak)	Yes (weak)	Yes (strong)	Yes (strong)	Yes (weak)	Yes (weak)	Yes (strong)	Yes (strong)	No	Yes (strong)
3C signal in vivo	NA	Yes (strong)	No	Yes (weak)	Yes (strong)	Yes (strong)	Yes (weak)	Yes (strong)	Yes (strong)	Yes (weak)	NA	No	NA
Virome signal in vitro	Yes	Yes	Yes	Yes	Yes	Yes	Yes	Yes	No	Yes	Yes	Yes	Yes
Virome signal in vivo	No	Yes	No	Yes	Yes	Yes	No	Yes	Yes	Yes	Yes	Yes	Yes
Circularization signal	Yes	Yes	Yes	Yes	Yes	Yes	Yes	Yes	Yes	No	Yes	Yes	Yes
Packaging method (PhageTermVirome)/termini position (in vitro reads)	COS 5' Visual confirmation 36,423	COS 5' Visual confirmation 194	COS 5' Visual confirmation 351	COS 5' Visual confirmation 15,785	Uncertain Potential PAC	Uncertain Potential COS 5'	COS 3' Visual confirmation 76,891 (right signal weak)	DTR Short (478 bp) 27,277	Uncertain Potential PAC	COS 5' Visual confirmation 21,757 (left signal weak)	COS 5' Visual confirmation 24,325	COS 5' Visual confirmation 28,255	COS 5' Visual confirmation 2483
Proposed morphology (vContact2)	Siphoviridae		Myoviridae	Myoviridae	Siphoviridae	Siphoviridae	Siphoviridae	Siphoviridae	Siphoviridae	Myoviridae		Siphoviridae	Siphoviridae

Gray columns indicate candidate prophages detected by Hi-C (dark gray = agreement between Hi-C and virome prophage coordinates, light gray = disagreement between Hi-C and virome prophage coordinates). Stars aside prophage name under the graph indicate phages described by Zünd et al

The comparison of the virome signal from the in vivo samples showed minimal differences between the two mice at each time point. Importantly, the same 10 prophages were induced in all four in vivo samples, demonstrating that the virome of the OMM^12^ mice is particularly stable between individuals and over time. The comparison between the two conditions showed that three prophages were induced exclusively in vitro and one exclusively in vivo. Moreover, the switch of *E. clostridioformis* and *C. innocuum* prophages activation detected by Hi-C was confirmed by virome sequencing. As previously described from a long-term metagenomic analysis of the OMM^12^ consortium [26], we also observed different hotspots of polymorphisms as well as some deletions in the genomes of the different induced prophages, notably in the prophage of *B. caecimuris* I48.

To further characterize the virome of OMM^12^ mice, we first sequenced additional samples spiked with known concentrations of phages [69]. This led us to estimate that OMM^12^ fecal samples contain around 10^8^ VLP/g of feces (Methods). Second, we searched for free viruses from reads that did not match OMM^12^ genomes to perform assemblies with MEGAHIT [49] and SPAdes [55] (~ 2–4% of the reads). However, this resulted in only five contigs of a size superior to 5 kb (Supplementary Table 3). BLAST analysis, using RefSeq database, of these contigs revealed that they correspond to a portion of induced prophages of *B. caecimuris* or *C. innocuum* and thus likely result from alignment errors (Supplementary Table 4). The unmapped reads were also used for taxonomic annotation using Kaiju [52] (Supplementary Fig. 7). Most reads (99.8%) were annotated as OMM^12^ community bacteria. The remaining 0.2% of reads annotated as viral were scattered among a variety of viral phyla, showing that no specific virus was identified. Third, we explored the presence of ssDNA phages [70] (Methods), known to be abundant in the mammalian gut virome [71], but found none. Therefore, the OMM^12^ gut harbors a stable population of induced prophages and neither virulent DNA phages nor eukaryotic DNA viruses.

We therefore demonstrated that the analysis of Hi-C data is relevant and reliable to distinguish between functional and cryptic prophages and to refine phage coordinates. We also show that the OMM^12^ mice provide a suitable in vivo environment to study the dynamics of intestinal prokaryotic and eukaryotic DNA viruses.

### In-depth analysis of induced prophages

The above 13 induced prophages were named after French castles, and their genomes were analyzed in detail (Fig. 5b and Table 2). First, we looked for a circularization signal, in the virome sample reads (Methods). Detected for 12 out of 13 prophages, this signal led us to precisely map the prophage coordinates. Second, using those exact coordinates, we annotated the phage genomes using the PHROG database [42] (Methods). A putative function was assigned to at least 25% and up to 60% of the genes including in all genomes the terminase and/or portal proteins, two hallmark proteins of phage genomes (Fig. 5b). Integrases were also frequently found, in agreement to the temperate lifestyle of these phages. Third, PhageTermVirome [44] was successfully used for 10 phages to identify their packaging method and termini (Table 2). Finally, we searched for homologs using BLAST against the nucleotide (nt) database, but only distant homologs were identified, showing that these prophages are largely new. Thus, we used vContact2 [45] to generate shared protein networks and identify the groups of phages that most closely relate to these 13 phages. While only a few high-quality RefSeq genomes clustered closely with the OMM^12^ prophages, many metagenomic phage sequences belonging to the Cenote Human Virome Database (CHVD) [47] closely clustered with them (Supplementary Fig. 8). This clustering allowed us to predict their possible morphology (Table 2). Overall, these 13 induced OMM^12^ phages represent newly characterized viruses of the intestinal microbiota, for which there is a sufficient basis for the creation of the new viral clades. Their annotation will notably contribute to lower the “viral dark matter” of metagenomics studies.

## Discussion

In the present study, we combined Hi-C and virome sequencing to dissect the chromosome architecture of a synthetic bacterial community representative of the murine gut microbiota, investigate their variations and identify its associated induced prophages in both in vitro and in vivo conditions. Most of the Hi-C studies using culture techniques have so far focused on model bacteria encompassing mainly representative of the proteo- and actinobacteria and were performed exclusively on in vitro grown cultures [59, 61, 72, 73]. The Hi-C results from the 12 non-model bacteria that belong to the major phylum of the mammals’ gut confirmed the conservation of the ParABS system and the condensins in the organization and the dynamics of bacterial chromosome architectures. On the other hand, the exact role of the Smc-ScpAB complex remains puzzling as some species encoding for this complex do not display a strong opposite diagonal (*L. reuteri* and *E. faecalis*) in their contact maps. It suggests that this complex may not be functional or is involved in a novel way that does not necessarily lead to an alignment of the chromosomal arms. Moreover, we also detected for *B. caecimuris* and *T. muris* a weak signal in the opposite diagonal while no clear homologs of condensins could be characterized in their genomes. As it is thought that condensins are essential actors of chromosome organization [66], our results suggest that other condensin-related proteins remain to be identified in bacteria. Taken together, we uncovered a diversity of architecture and genomic organization by studying non-model bacteria and linking 3D organization with the physiological state (in vitro vs in vivo growth).

We also found that the global chromosome folding of the 12 bacteria is preserved in the gut compared to in vitro condition with, still, notable differences at the local scale. Some structures appear less pronounced in the contact matrices obtained with fecal samples, and the overall ratio of short- and long-range interactions appear to change between the two conditions. This result is likely due to differences in the growth conditions imposed by the gut environment and demonstrates its high impact on bacterial physiology. Such observation is in line with contact maps obtained from stationary phase cultures that exhibit less pronounced signals in the main diagonal but higher short-range interactions compared to exponential growth conditions [7]. However, in our data, the ratio of the ori/ter coverage indicates that bacteria are dividing. Future studies combining Hi-C with RNAseq will provide a better understanding of the link between chromosome 3D architecture, transcriptional activity, and the physiological state of the bacteria in the intestinal tract. Given the stability over time of the observed structures, the OMM^12^ mice represent a very promising model to study the genome dynamics of intestinal microbial communities at a very high resolution.

The prophage activity in host bacteria is generally studied either by qPCR or by exploiting sequencing reads from enriched virions or fecal samples and using phage reference genomes [38]. Here, we leverage Hi-C with virome data to propose a new way to characterize potentially active and functional prophages. In the present study, we used an insulation score to detect a CID-like pattern, sometimes associated with a loop, for 16 prophages among the 44 candidates predicted from genome analysis [8, 19]. Among these 16 prophages, we found that 11 produced particles in vitro and/or in vivo*.* We also found that only two induced prophages did not display a CID-like structuration. Of particular interest is the use of the Hi-C data and computational tools to detect CIDs (like insulation score or directional index) to refine the borders of the prophage loci predicted by state-of-the-art tools. Borders of CIDs in bacteria have been correlated with the presence of long and/or highly transcribed genes as well as with the presence of architectural proteins [6–8, 13, 59]. The presence of such structuration at functional prophages loci indicates the presence of topological constraints applied by proteins and/or transcriptional activity that could potentially regulate prophage activities. Moreover, our approach also offers the possibility to detect circular or loop signals that can help detect prophage activation and could as well allow the characterization of their replication and packaging strategy.

We found seven additional phages induced in vitro and/or in vivo compared to the six mitomycin C-induced prophages previously reported from individual cultures [38]. This confirms that the SOS response induced by DNA damage is not a universal way of inducing prophages [74]. In addition, some prophages were differentially induced between the two conditions, with three only induced in vitro and one only induced in vivo. The case of the prophage I48-pp-2.969 of *B. caecimuris* is very striking, while we could detect it in the VLPs in the different samples, its tridimensional organization presents important differences between in vitro and in vivo conditions suggesting a different physiological state. These differences could be driven by the gut environment or the contact with the other bacteria. Previous studies have documented particular prophage dynamics and evolution in various conditions [75, 76] and more specifically in the gut of mice [26, 74, 77–79], but the impact of the intestinal environment on prophage induction remained poorly understood. Here, we showed that the resident viral community of the OMM^12^ mice is stable over time, being uniquely constituted of temperate phages, which is consistent with the high stability of the bacteriome in these mice [23]. This is also coherent with the current appreciation of the human virome, which is thought to have a minimal intra-personal variability [80]. Analysis 1 year apart confirmed the presence of polymorphisms and potentially positive selection over time. A comparison of our different virome samples shows that the prophage population slowly evolved overtime potentially through selection at the bacterial population level as induced phages likely cannot reinfect their hosts, and therefore, likely cannot propagate those mutations. The detection of a deletion as well as signatures of positive selection in 2 genes within the active prophage of *B. caecimuris* is again interesting and could reflect different phenomena such as active lysogeny [75] or morons [81]. Our results advocate for the relevance of the OMM^12^ model to study intestinal virus dynamics and their evolution through time and changing environments.

This first characterization of the viral community of gnotobiotic animals showed that the 13 phages are also all distantly related to well-characterized phages and will lead to the creation of new viral clades, which is a significant step towards a better understanding of gut viral communities. In addition, we found that the OMM^12^ mice do not carry any eukaryotic intestinal DNA virus. Such viruses are part of the usual mouse microbiota [82, 83] and have been shown to contribute to the development of immunity [84, 85]. Their absence might have consequences on the maturation of the immune system but also offers opportunities to study this process for individual viruses (pathogens or commensals) in the presence of a defined intestinal microbiota. In addition, we did not detect virulent phages in the virome samples. The absence of virulent phages and eukaryotic viruses is however not surprising considering the stringent conditions in which these mice are bred.

## Conclusion

Altogether, these data, obtained with a gnotobiotic murine model, have demonstrated the benefit of the Hi-C approach to not only unveil novel chromosome architectures but also to detect the dynamic induction of prophages. This extensive characterization of the dynamic variations of the bacteriome and the virome of an intestinal community provides a path to move microbiota studies from correlation to causality. Combination with other technologies will allow us to better understand the link between 3D architectures and the physiological states of prophages in the mammal intestinal tract.

## Supplementary Information

**Additional file 1:** **Supplementary Figure 1a and 1b**. Contact map of each bacterial strain of the OMM^12^ consortium obtained from in vitro cultures. **Supplementary Figure 2.** Re-assembly of B. animalis, F. plautii, and B. caecimuris. **Supplementary Figure 3.** Signal of the secondary diagonals for the different bacteria of the OMM^12^ consortium. **Supplementary Figure 4.** Comparison of the contact maps (in vitro vs. in vivo) for the six most abundant bacteria. **Supplementary Figure 5.** Hierarchical clustering of the different Hi-C replicates for the different bacteria of the OMM^12^ consortium using the software HiCrep. **Supplementary Figure 6a and 6b.** Contact maps of functional prophage candidates (+/- 50kb). **Supplementary Figure 7.** Krona representation of the Kaiju annotation of the reads not mapping on the OMM12 25 strains’ genomes. **Supplementary Figure 8.** Viral clustering of the 13 induced phages using vContact2. **Supplementary Table 1.** Genomic libraries generated. **Supplementary Table 2.** Genbank accession numbers of the OMM12 bacteria genomes. **Supplementary Table 3.** Metrics of the assemblies obtained with virome reads that did not map on the OMM12 strains. **Supplementary Table 4.** Blast results of the contigs obtained by assembling non-mapping reads.

## Data Availability

Sequence data as well as new bacterial genomes have been deposited in the NCBI under the BioProject number PRJNA831628. Phage genomes are available using the following project number: PRJNA831646. The code used in the present study can be found at the following address https://github.com/abignaud/oligomm_analysis
