# Supplementary material for: Chromosome folding and prophage activation reveal specific genomic architecture for intestinal bacteria
Source: Microbiome. 2023 May 19;11:111. doi: 10.1186/s40168-023-01541-x (PMC10197239; doi:10.1186/s40168-023-01541-x)
Supplement: Supplementary file 2 — Additional file 1: Supplementary Figure 1a and 1b. Contact map of each bacterial strain of the OMM12 consortium obtained from in vitro cultures. Supplementary Figure 2. Re-assembly of B. animalis, F. plautii, and B. caecimuris. Supplementary Figure 3. Signal of the secondary diagonals for the different bacteria of the OMM12 consortium. Supplementary Figure 4. Comparison of the contact maps (in vitro vs. in vivo) for the six most abundant bacteria. Supplementary Figure 5. Hierarchical clustering of the different Hi-C replicates for the different bacteria of the OMM12 consortium using the software HiCrep. Supplementary Figure 6a and 6b. Contact maps of functional prophage candidates (+/- 50kb). Supplementary Figure 7. Krona representation of the Kaiju annotation of the reads not mapping on the OMM12 25 strains’ genomes. Supplementary Figure 8. Viral clustering of the 13 induced phages using vContact2. Supplementary Table 1. Genomic libraries generated. Supplementary Table 2. Genbank accession numbers of the OMM12 bacteria genomes. Supplementary Table 3. Metrics of the assemblies obtained with virome reads that did not map on the OMM12 strains. Supplementary Table 4. Blast results of the contigs obtained by assembling non-mapping reads. [file 40168_2023_1541_MOESM1_ESM.pdf]

SUPPLEMENTARY DATA

Chromosome folding and prophage activation reveal specific genomic architecture for intestinal bacteria

Authors

Quentin LAMY-BESNIER[1,2], Amaury BIGNAUD[2,3], Julian R. GARNEAU[4], Marie TITECAT[1], Devon CONTI[1,2,3], Alexandra VON STREMPEL[5], Marc MONOT[4], Bärbel STECHER[5,6], Romain KOSZUL[2], Laurent DEBARBIEUX[1], Martial MARBOUTY[2]

Supplementary Figure 1a and 1b: Contact map of each bacterial strain of the OMM^{12} consortium obtained from *in vitro* cultures.
Supplementary Figure 2: Re-assembly of *B. animalis*, *F.* plautii, and *B. caecimuris*.
Supplementary Figure 3: Signal of the secondary diagonals for the different bacteria of the OMM^{12} consortium.
Supplementary Figure 4: Comparison of the contact maps (*in vitro* vs. *in* vivo) for the six most abundant bacteria.
Supplementary Figure 5: Hierarchical clustering of the different Hi-C replicates for the different bacteria of the OMM^{12} consortium using the software HiCrep.
Supplementary Figure 6a and 6b: Contact maps of functional prophage candidates (+/- 50 kb).
Supplementary Figure 7: Krona representation of the Kaiju annotation of the reads not mapping on the OMM^{12} strains' genomes.
Supplementary Figure 8: Viral clustering of the 13 induced phages using vContact2.

Supplementary Table 1: Genomic libraries generated.
Supplementary Table 2: Genbank accession numbers of the OMM12 bacteria genomes.
Supplementary Table 3: Metrics of the assemblies obtained with virome reads that did not map on the OMM12 strains.
Supplementary Table 4: Blast results of the contigs obtained by assembling non-mapping reads.

Supplementary Figure 1a and 1b: Contact map of each bacterial strain of the OMM[12] consortium obtained from *in vitro* cultures.

Each contact map is represented with associated genomic information: localization of *parS* sites (green), tRNA (blue) and rRNA (red) on top with below prophage annotation (blue), coverage, GC content and GC skew.

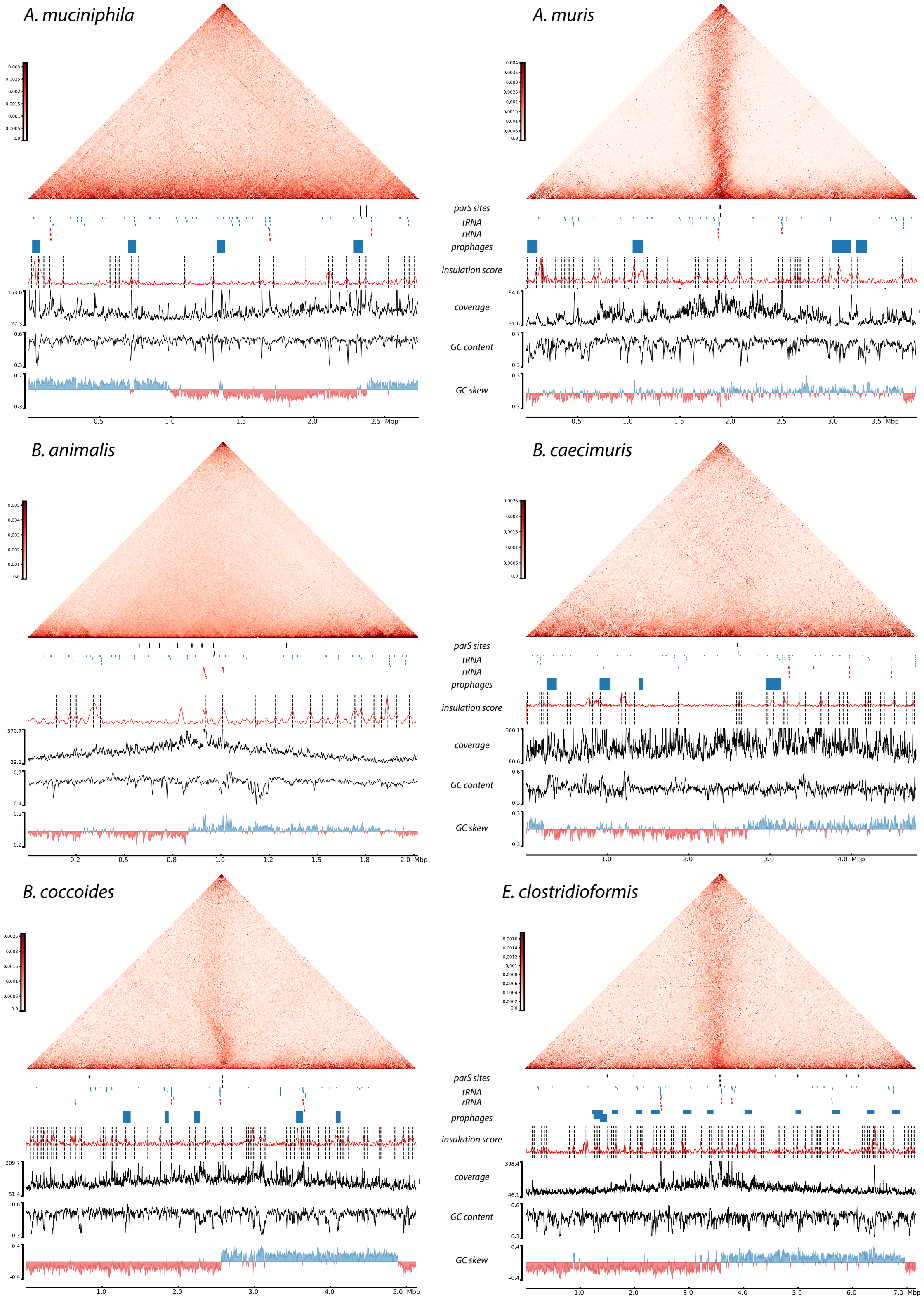

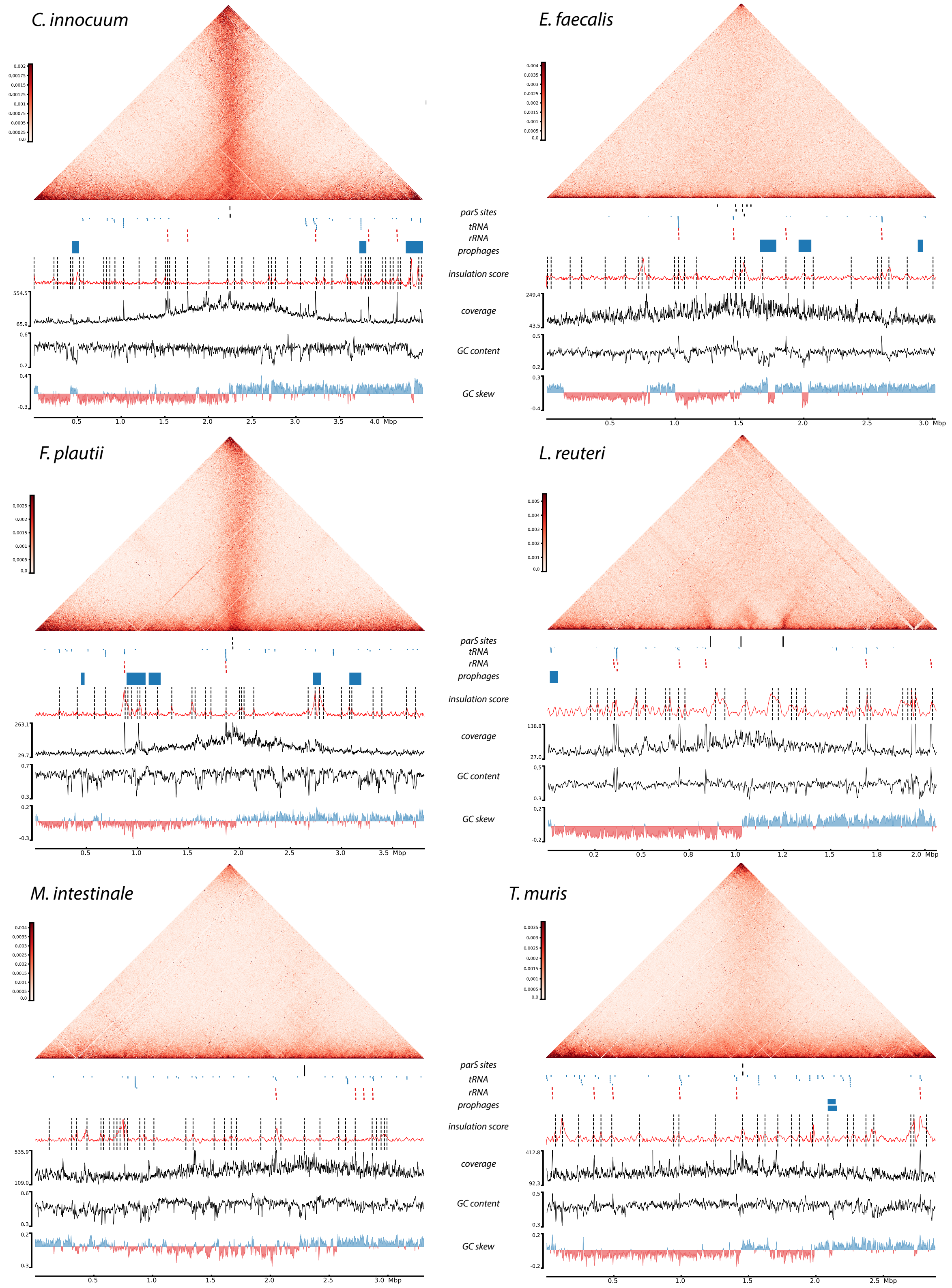
C. innocuum
E. faecalis
F. plautii
L. reuteri
M. intestinale
T. muris
parS sites
tRNA
rRNA
prophages
insulation score
coverage
GC content
GC skew
Mbp

Supplementary Figure 2: Re-assembly of *B. animalis*, *F.* plautii, and *B. caecimuris*. The contact maps are shown before (left) and after (right) Hi-C-based re-assembly.

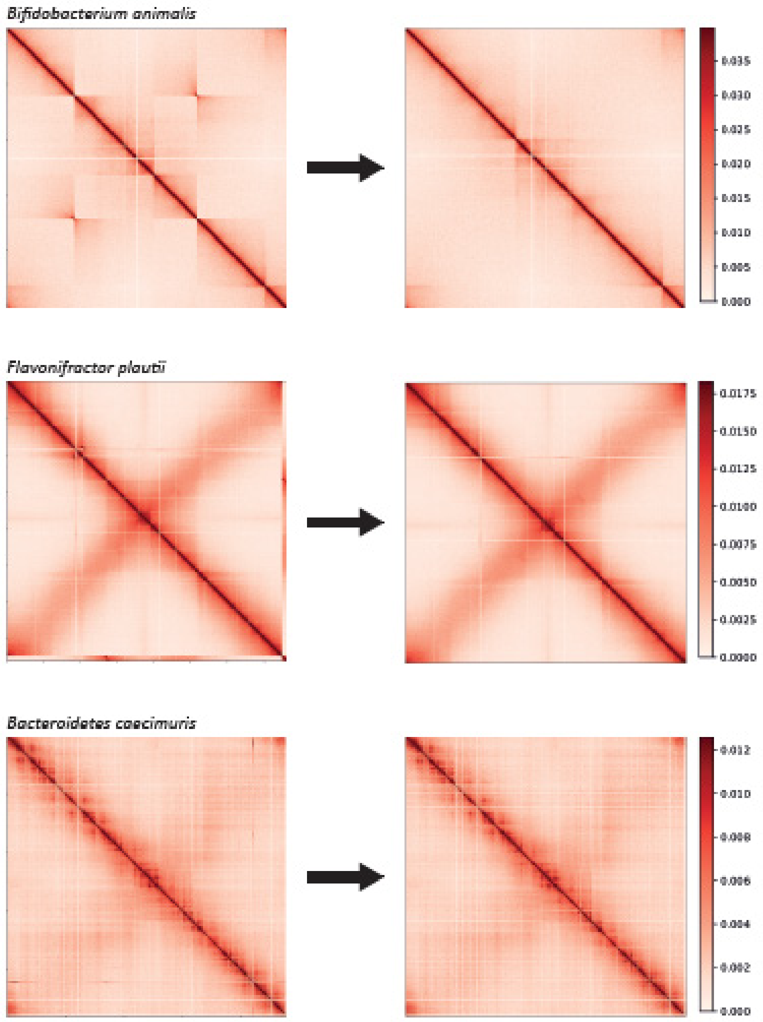

Supplementary Figure 3: Signal of the secondary diagonals for the twelve bacteria of the OMM[12] consortium grown *in vitro*.

Plots are centered on *ori*. Localisation of *parS* sites are indicated as red dashed lines. Red stars indicate the presence of several *parS* sites in the same 5 kb window.

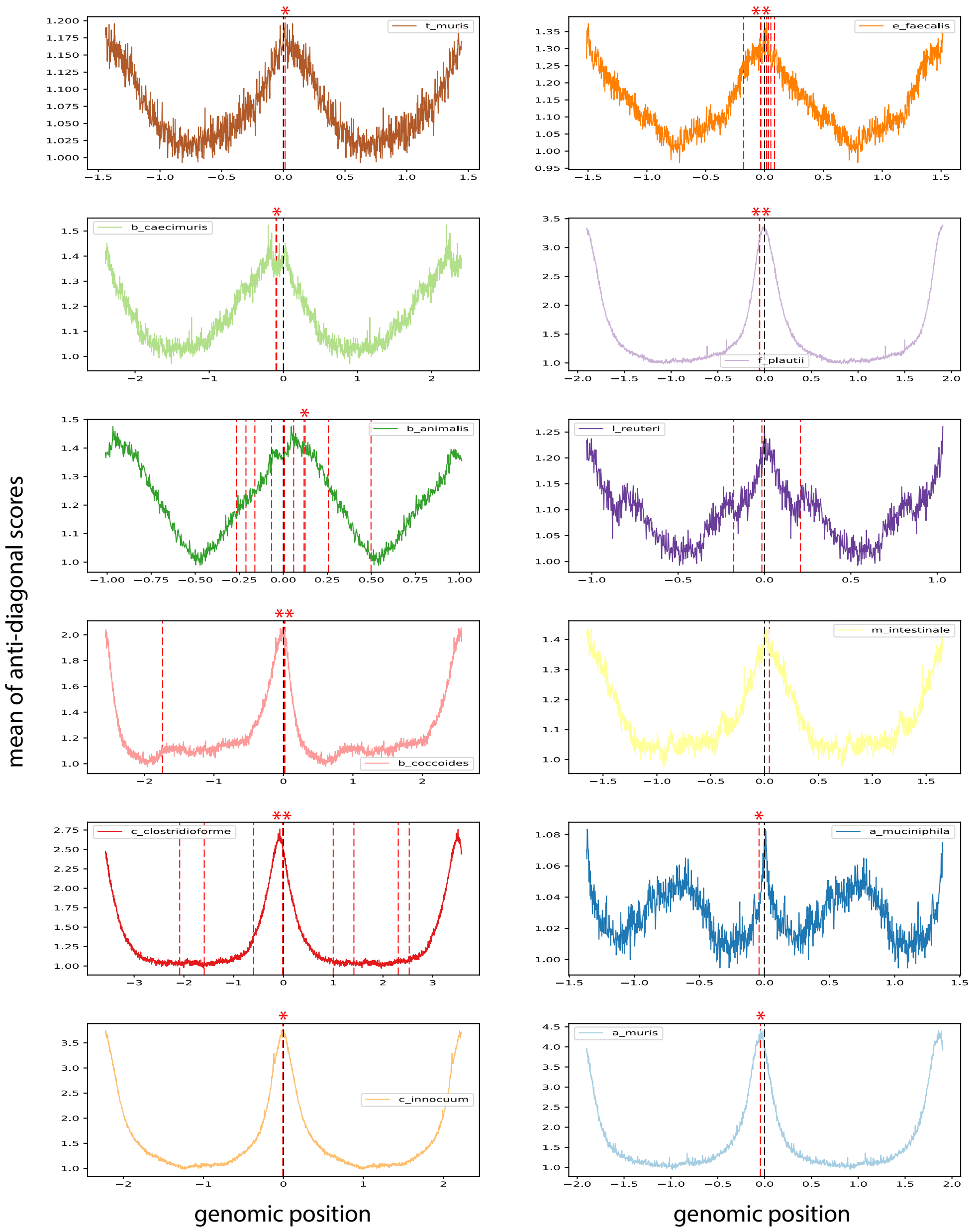

Supplementary Figure 4: Comparison of the contact maps (*in vitro* vs. *in vivo*) for the six most abundant intestinal bacteria in OMM[12] mice.

a. *In vitro* (5 kb bin), *in vivo* (5 kb bin) and ratio (Log2; 10 kb bin) of contact maps (*in vitro vs. in vivo*) obtained for the six most abundant bacteria in OMM[12] mice. Specific annotations are indicated under matrices. Origin of replication are indicated by dashed black lines. **b.** The three matrices (*in vitro* (up), *in vivo* 2019 (middle) and *in vivo* 2020 (bottom)) are shown on top of each other.

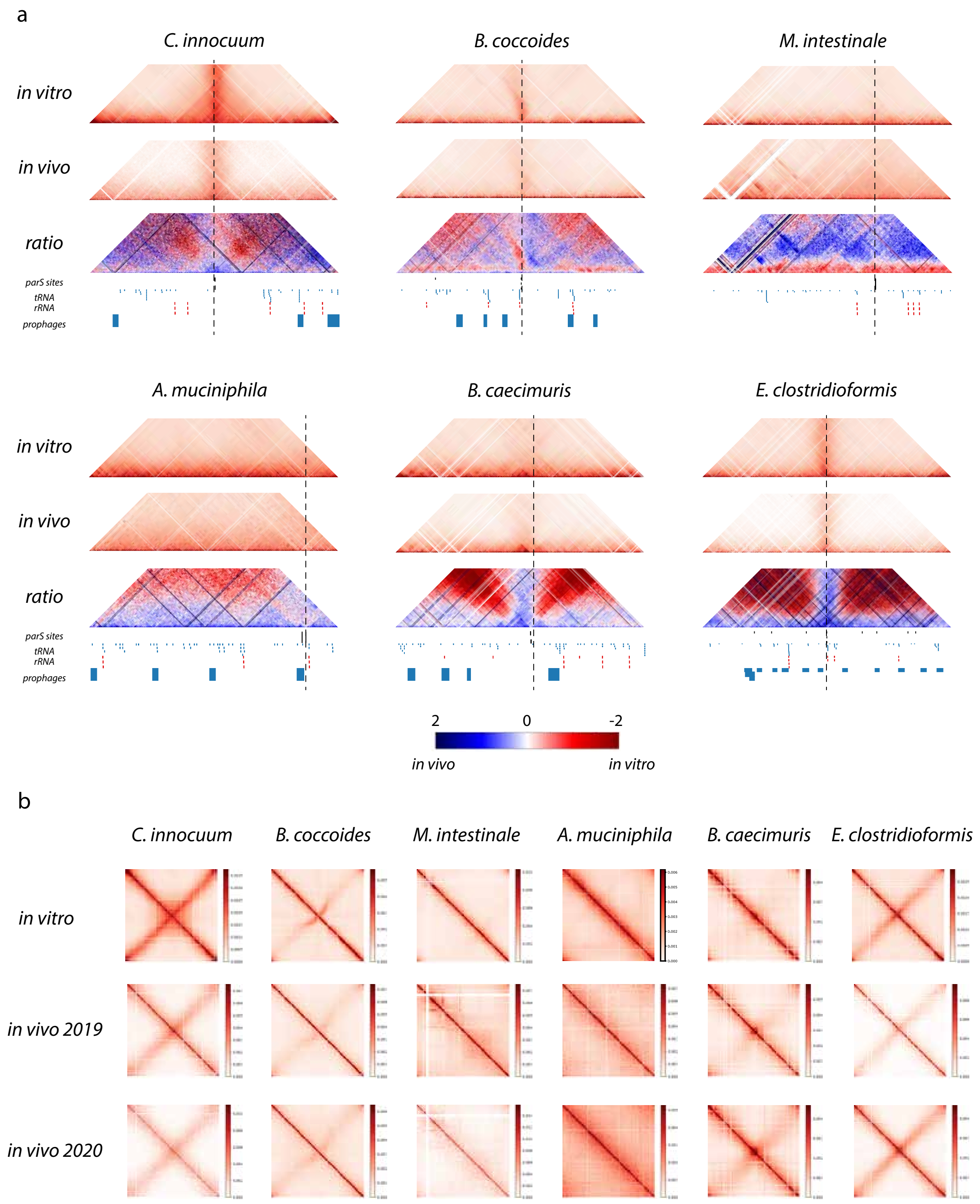

Supplementary Figure 5: Hierarchical clustering of the different Hi-C replicates for the different bacteria of the OMM[12] consortium using the software HiCrep.

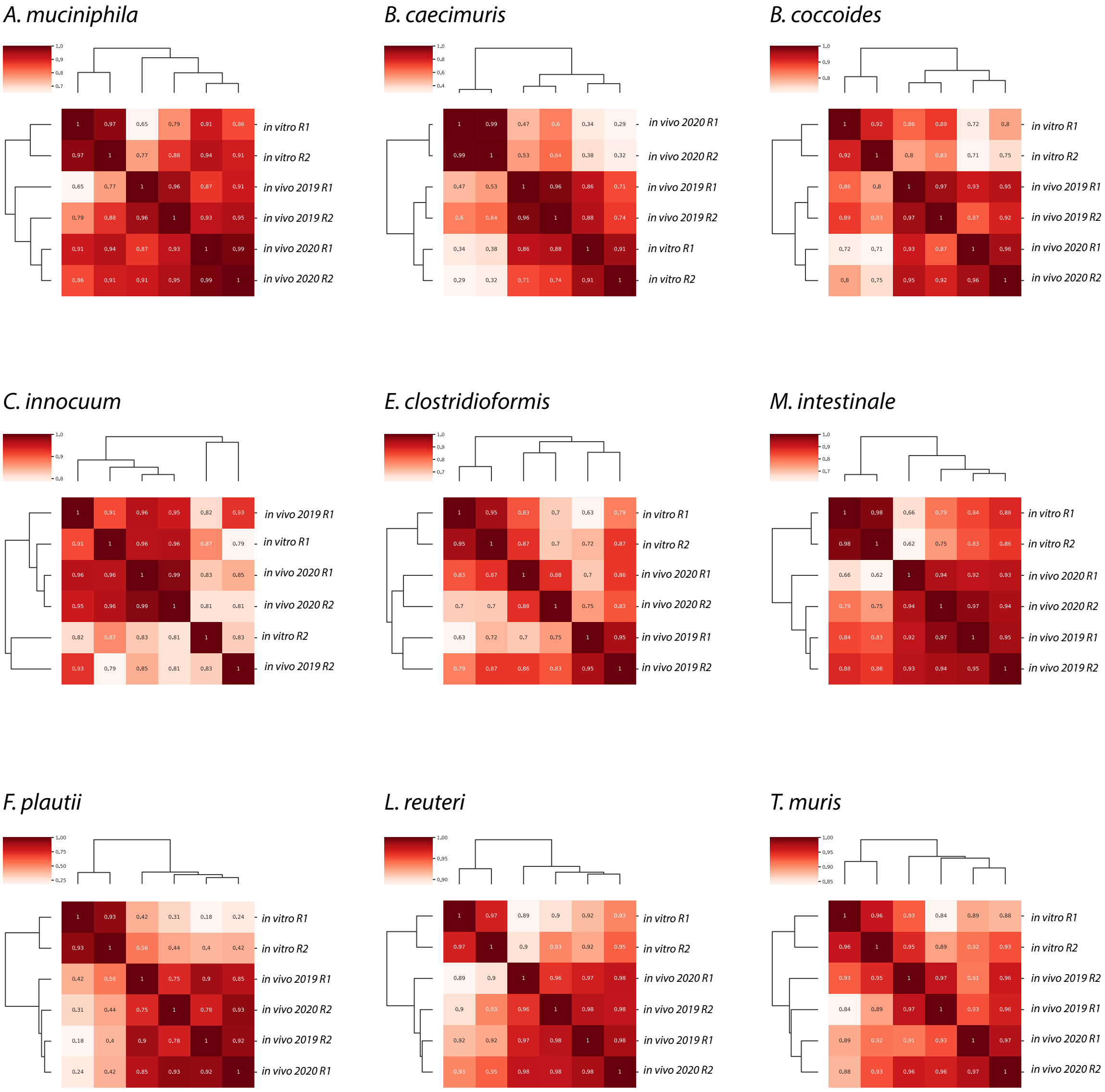

Supplementary Figure 6a and 6b: Contact maps of functional prophage candidates (+/- 50 kb).

Genomic coordinates are indicated above contact maps while Hi-C coverage as well as virome data are indicated under. Dashed lines indicate Hi-C refinement of prophage coordinates. Blue and red regions indicate, respectively, chromosome and predicted prophages.

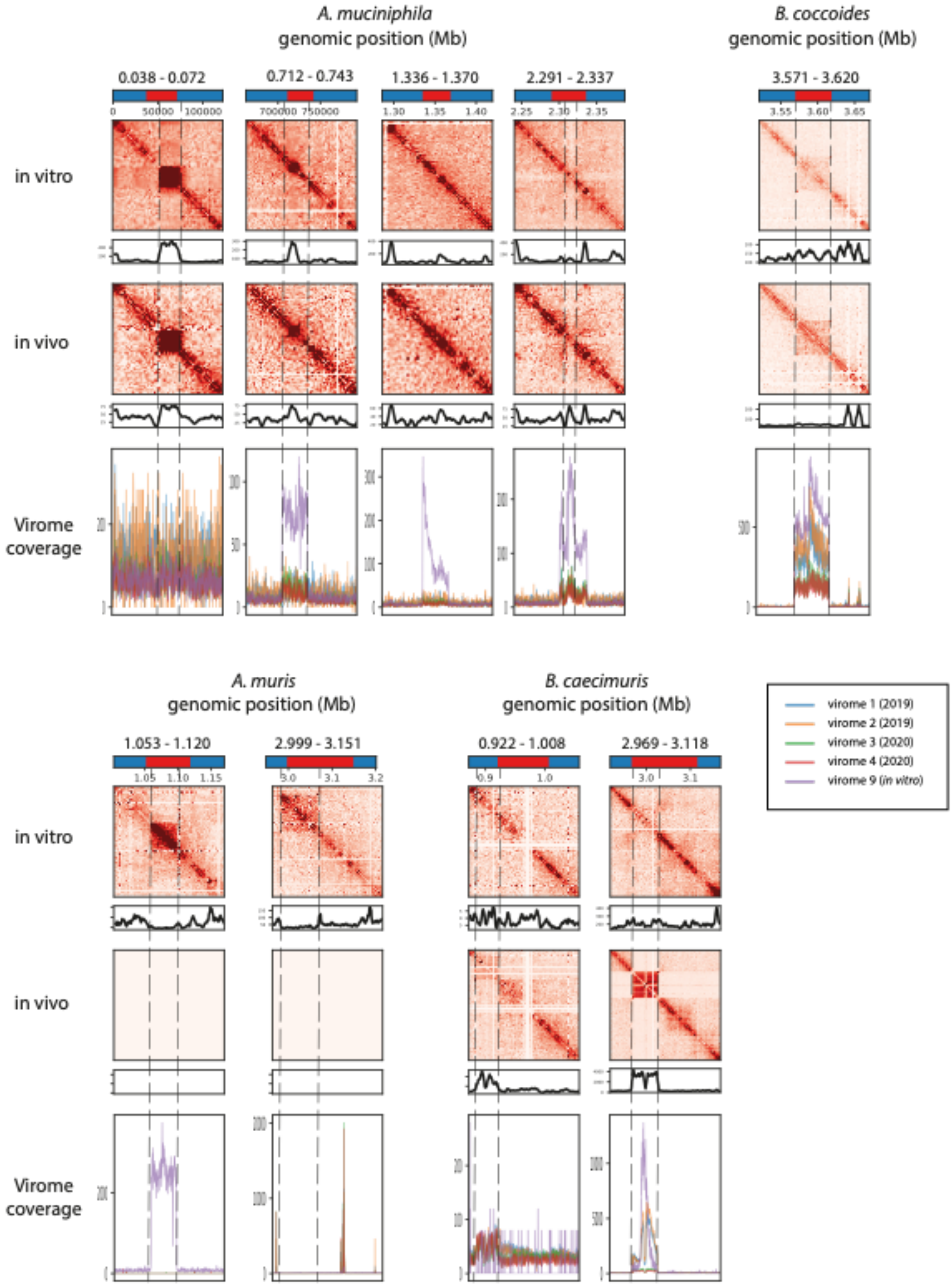

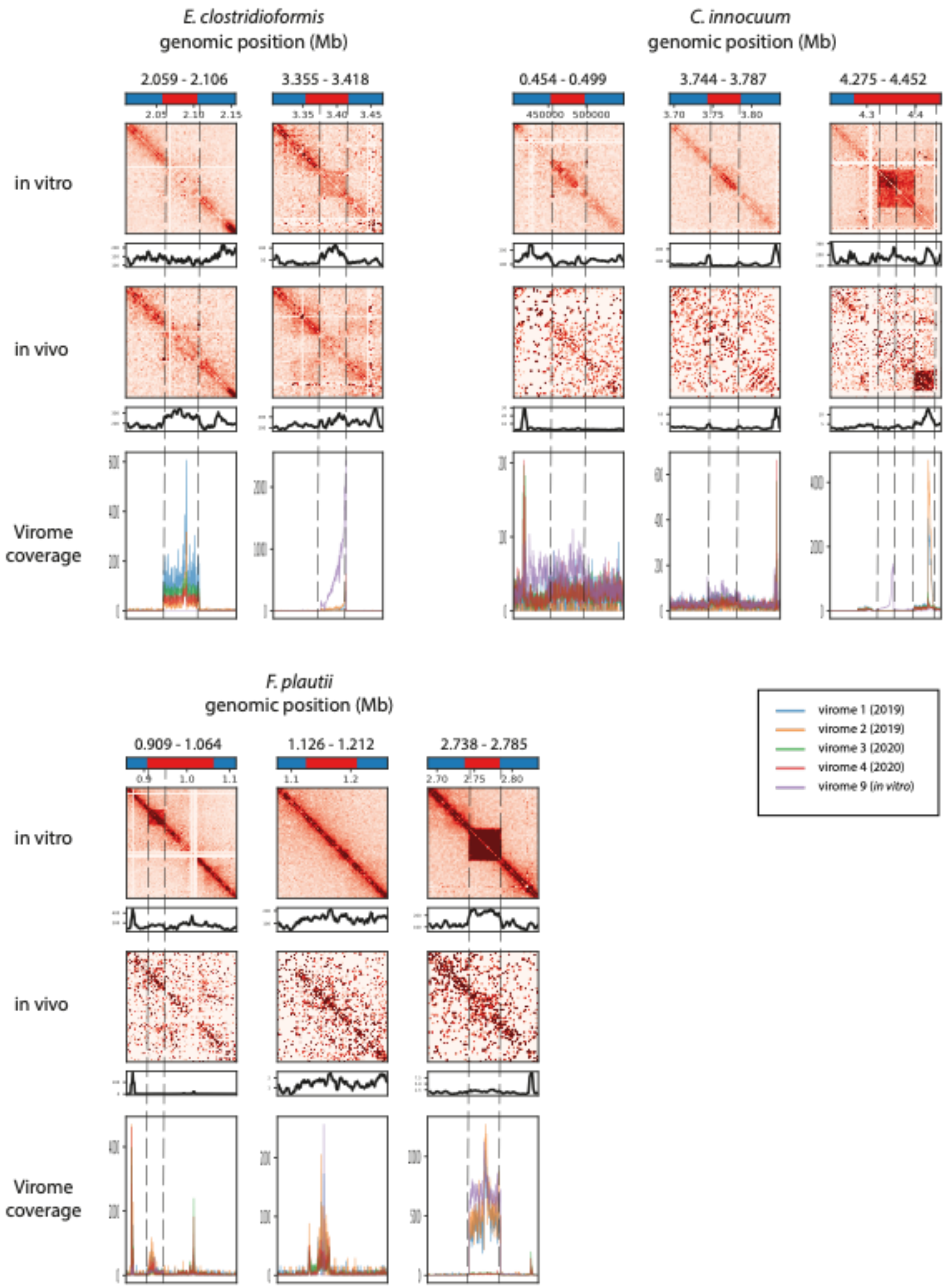
E. clostridioformis
genomic position (Mb)
2.059 - 2.106
3.355 - 3.418
C. innocuum
genomic position (Mb)
0.454 - 0.499
3.744 - 3.787
4.275 - 4.452
in vitro
in vivo
Virome coverage
F. plautii
genomic position (Mb)
0.909 - 1.064
1.126 - 1.212
2.738 - 2.785
in vitro
in vivo
Virome coverage
virome 1 (2019)
virome 2 (2019)
virome 3 (2020)
virome 4 (2020)
virome 9 (in vitro)

Supplementary Figure 7: Krona representation of the Kaiju annotation of the reads not mapping on the OMM^{12} strains' genomes.

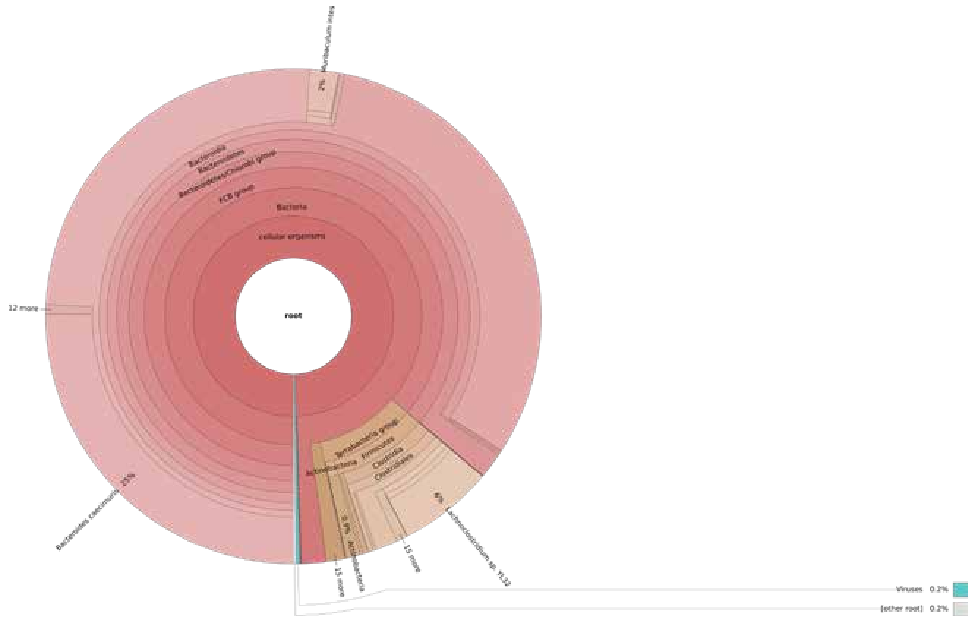

Supplementary Figure 8: Viral clustering of the 13 induced prophages using vConctact2. **a.** Viral cluster analysis with vConTACT2 using a gene-sharing network. The analysis was performed using genomes from the ViralRefSeq V.201 (red nodes) and CHVD (grey nodes) reference databases. Nodes for OMM[12] prophages were colored according to their respective host. **b.** Close-up view of the 13 OMM[12] inducible prophages and their direct (first level) neighbors in the network.

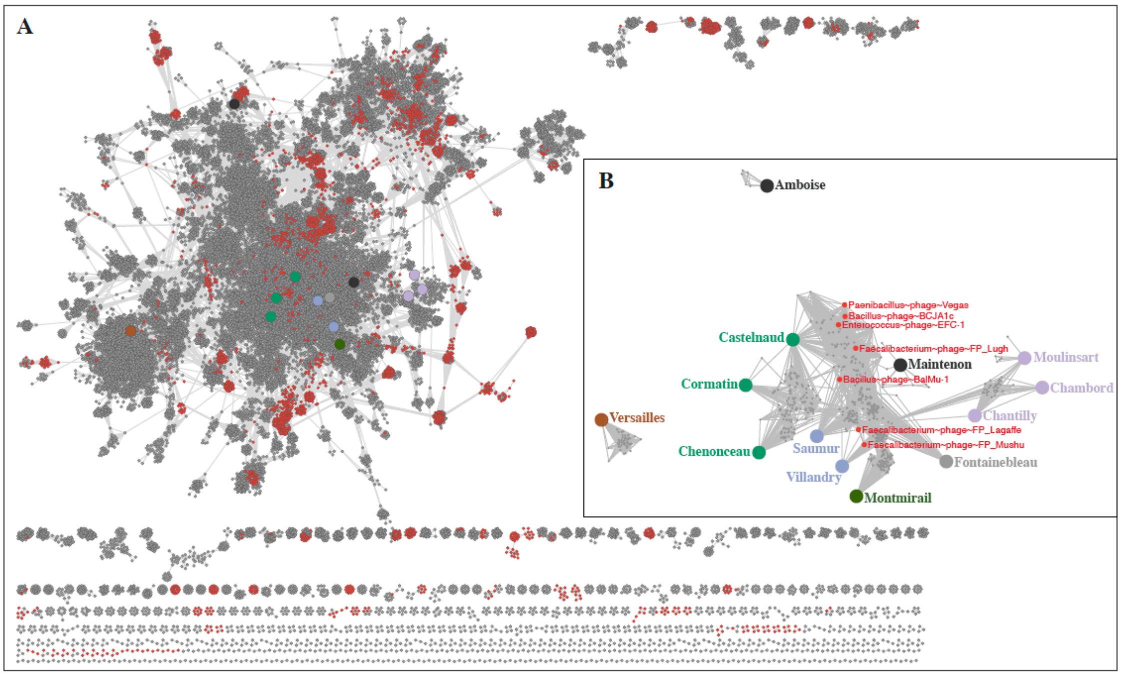

Supplementary Table 1: Genomic libraries generated.

a: libraries Virome_5 and Virome_6 were prepared with Accel-NGS™ 1S Plus DNA Library Kit, and thus contain accurate data for both ssDNA and dsDNA viruses. b: spike 1 was composed of 10^8 PFU/mL of phages CLB_P1, CLB_P2, CLB_P3 and M13. c: spike 2 was composed of 10^7 PFU/mL of phages CLB_P1, CLB_P2, CLB_P3 and M13.

**type**	lib id	genome	species	conditions	cage	sex	sampling date	raw reads (paired-end)	Accession number
**Hi-C *in vitro***	OMM5	I46	*C.innocuum*	Lab	NA	NA	march-21	41 561 382	SRX15328653
	OMM5_rep	I46	*C.innocuum*	Lab	NA	NA	July-21	10 688 910	SRX15328654
	OMM6	YL58	*B.coccoides*	Lab	NA	NA	march-21	21 485 845	SRX15328665
	OMM6_rep	YL58	*B.coccoides*	Lab	NA	NA	July21	2 257 624	SRX15328676
	OMM7	I48	*B.caecimuris*	Lab	NA	NA	march-21	93 139 653	SRX15328684
	OMM7_rep	I48	*B.caecimuris*	Lab	NA	NA	July-21	61 639 162	SRX15328685
	OMM8	YL27	*M.intestinale*	Lab	NA	NA	march-21	31 275 362	SRX15328686
	OMM8_rep	YL27	*M.intestinale*	Lab	NA	NA	July-21	9 298 553	SRX15328687
	OMM9	YL44	*A.muciniphila*	Lab	NA	NA	march-21	40 820 347	SRX15328688
	OMM9_rep	YL44	*A.muciniphila*	Lab	NA	NA	July-21	7 062 251	SRX15328689
	OMM10	YL32	*C.clostridioforme*	Lab	NA	NA	march-21	62 518 175	SRX15328655
	OMM10_rep	YL32	*C.clostridioforme*	Lab	NA	NA	July-21	6 408 840	SRX15328656
	OMM11	KB1	*E.faecalis*	Lab	NA	NA	march-21	12 250 166	SRX15328657
	OMM11_rep	KB1	*E.faecalis*	Lab	NA	NA	July-21	11 076 026	SRX15328658
	OMM12	YL31	*F.plautii*	Lab	NA	NA	march-21	10 544 208	SRX15328659
	OMM12_rep	YL31	*F.plautii*	Lab	NA	NA	July-21	17 539 144	SRX15328660
	OMM13	I49	*L.reuteri*	Lab	NA	NA	march-21	9 363 639	SRX15328661
	OMM13_rep	I49	*L.reuteri*	Lab	NA	NA	July-21	4 516 936	SRX15328662
	OMM14	YL2	*B.animalis*	Lab	NA	NA	march-21	7 526 155	SRX15328663
	OMM14_rep	YL2	*B.animalis*	Lab	NA	NA	July-21	4 669 716	SRX15328664
	OMM15	YL45	*T.muris*	Lab	NA	NA	march-21	8 827 292	SRX15328666
	OMM15_rep	YL45	*T.muris*	Lab	NA	NA	July-21	18 422 688	SRX15328667
	OMM16	KB18	*A.muris*	Lab	NA	NA	march-21	7 757 787	SRX15328668
	OMM16_rep	KB18	*A.muris*	Lab	NA	NA	July-21	9 033 311	SRX15328669
**Hi-C *in vivo***	OMM1	mix	microbiota	microbiota	3	male	sept-19	101 182 905	SRX15328670
	OMM2	mix	microbiota	microbiota	4	female	sept-19	115 105 053	SRX15328671
	OMM3	mix	microbiota	microbiota	1	male	may-20	72 638 739	SRX15328672
	OMM4	mix	microbiota	microbiota	2	female	may-20	94 694 874	SRX15328673
**Virome**	Virome1	mix	dsDNA	microbiota	4	female	sept-19	6 443 793 (2x35)	SRX15328674
	Virome2	mix	dsDNA	microbiota	3	male	sept-19	4 957 507 (2x35)	SRX15328675

	Virome3	mix	dsDNA	microbiota	1	male	may-20	108 807 445 (2x35 & 2x150)	SRX15328677
	Virome4	mix	dsDNA	microbiota	2	male	may-20	119 010 486 (2x35 & 2x150)	SRX15328678
	Virome5	mix	ssDNA[a] + spike 1[b]	microbiota	breeding	mix	march-21	1 056 335 (2x150)	SRX15328679
	Virome6	mix	ssDNA[a] + spike 1[b]	microbiota	breeding	mix	march-21	2 763 300 (2x150)	SRX15328680
	Virome7	mix	dsDNA + spike 2[c]	microbiota	breeding	mix	march-21	5 174 852 (2x150)	SRX15328681
	Virome8	mix	dsDNA + spike 2[c]	microbiota	breeding	mix	march-21	4 339 389 (2x150)	SRX15328682
	Virome9	mix (in vitro)	dsDNA	microbiota	breeding	NA	oct-21	6 979 488 (2x150)	SRX15328683

Supplementary Table 2: Genbank accession numbers of the OMM12 bacteria genomes.

Genus	Species	Strain	GenBank assembly accession
**akkermansia**	**muciniphila**	**YL44**	**GCA_016697425.1**
**acutalibacter**	**muris**	**KB18**	**GCA_016697365.1**
**bifidobacterium**	**animalis**	**YL2**	**GCA_023278655.1**
**bacteroides**	**caecimuris**	**I48**	**GCA_023277905.1**
**blautia**	**pseudococcoides**	**YL58**	**GCA_016696745.1**
**clostridium**	**innocuum**	**I46**	**GCA_016697325.1**
**enterocloster**	**clostridoformis**	**YL32**	**GCA_016696785.1**
**enterococcus**	**faecalis**	**KB1**	**GCA_016696825.1**
**flavonifractor**	**plautii**	**YL31**	**GCA_023277885.1**
**limosilactobacillus**	**reuteri**	**I49**	**GCA_016697045.1**
**muribaculum**	**intestinale**	**YL27**	**GCA_016696845.1**
**turicimonas**	**muris**	**YL45**	**GCA_016696765.1**

Supplementary Table 3: Metrics of the assemblies obtained with virome reads that did not map on the OMM^{12} strains.
Both SPAdes and Megahit were used.

			SPAdes		Megahit	
Sample name	Sample info	Number of non mapping reads	Number of contigs	Number of contigs >5kb	Number of contigs	Number of contigs >5kb
#1	2019, 2x35	358 904	68	2	59	3
#2	2019, 2x35	467 489	46	2	50	5
#3-35	2020, 2x35	6 530 360	10766	1	8214	0
#3-150	2020, 2x150	791 870	1611	2	2075	0
#4-35	2020, 2x35	10 045 524	10241	0	5903	0
#4-150	2020, 2x150	471 135	3119	0	4320	0
merge	all above samples	18 665 282	14012	0	8721	0
merge #3	2020, 2x35+2x150	7 322 230	15107	0	5887	0
merge #4	2020, 2x35+2x150	10 516 659	11067	0	5903	0

Supplementary Table 4: Blast results of the contigs obtained by assembling non-mapping reads.

software	Contig name	Associated sample	Size	Best blast hit	Blast %coverage	Blast %id	Blast e-value
SPADES	CS1	#1 (2019)	24 646	B_caecimuris	100	99.90	0
	CS2	#1 (2019)	22 127	B_caecimuris	100	99.78	0
	CS3	#2 (2019)	53 027	B_caecimuris	100	99.8	0
	CS4	#2 (2019)	7 265	C. innocuum	100	100	0
	CS6	#3-150 (2020, 150)	6 639	B_caecimuris	100	99.95	0
	CS7	#3-150 (2020, 150)	6 369	B_caecimuris	100	99.76	0
	CS8	#4-150 (2020, 150)	5 854	B_caecimuris	100	99.79	0
MegaHit	CM1	#1 (2019)	33 237	B. caecimuris	100	99.84	0
	CM2	#1 (2019)	9 945	B. caecimuris	100	99.82	0
	CM3	#1 (2019)	6 574	B. caecimuris	99	99.71	0
	CM4	#2 (2019)	20 229	B. caecimuris	100	99.83	0
	CM5	#2 (2019)	9 690	B. caecimuris	100	99.96	0
	CM6	#2 (2019)	8 089	B. caecimuris	100	99.84	0
	CM7	#2 (2019)	6 839	B. caecimuris	99	99.9	0
	CM8	#2 (2019)	5 999	C. innocuum	100	100	0
